# Transcriptome Analysis of *Neisseria meningitidis* in Human Whole Blood and Mutagenesis Studies Identify Virulence Factors Involved in Blood Survival

**DOI:** 10.1371/journal.ppat.1002027

**Published:** 2011-05-05

**Authors:** Hebert Echenique-Rivera, Alessandro Muzzi, Elena Del Tordello, Kate L. Seib, Patrice Francois, Rino Rappuoli, Mariagrazia Pizza, Davide Serruto

**Affiliations:** 1 Novartis Vaccines and Diagnostics, Siena, Italy; 2 Genomic Research Laboratory, University of Geneva Hospitals (HUG), Geneva, Switzerland; Northwestern University Feinberg School of Medicine, United States of America

## Abstract

During infection *Neisseria meningitidis* (Nm) encounters multiple environments within the host, which makes rapid adaptation a crucial factor for meningococcal survival. Despite the importance of invasion into the bloodstream in the meningococcal disease process, little is known about how Nm adapts to permit survival and growth in blood. To address this, we performed a time-course transcriptome analysis using an *ex vivo* model of human whole blood infection. We observed that Nm alters the expression of ≈30% of ORFs of the genome and major dynamic changes were observed in the expression of transcriptional regulators, transport and binding proteins, energy metabolism, and surface-exposed virulence factors. In particular, we found that the gene encoding the regulator Fur, as well as all genes encoding iron uptake systems, were significantly up-regulated. Analysis of regulated genes encoding for surface-exposed proteins involved in Nm pathogenesis allowed us to better understand mechanisms used to circumvent host defenses. During blood infection, Nm activates genes encoding for the factor H binding proteins, fHbp and NspA, genes encoding for detoxifying enzymes such as SodC, Kat and AniA, as well as several less characterized surface-exposed proteins that might have a role in blood survival. Through mutagenesis studies of a subset of up-regulated genes we were able to identify new proteins important for survival in human blood and also to identify additional roles of previously known virulence factors in aiding survival in blood. Nm mutant strains lacking the genes encoding the hypothetical protein NMB1483 and the surface-exposed proteins NalP, Mip and NspA, the Fur regulator, the transferrin binding protein TbpB, and the L-lactate permease LctP were sensitive to killing by human blood. This increased knowledge of how Nm responds to adaptation in blood could also be helpful to develop diagnostic and therapeutic strategies to control the devastating disease cause by this microorganism.

## Author Summary

*Neisseria meningitidis* (Nm) is an exclusively human pathogen and a leading cause of bacterial meningitis and septicemia worldwide. Characterization of the bacterial transcriptome during host-pathogen interactions is a fundamental step for understanding the infectious processes of bacterial pathogens. Despite the severity of meningococcal sepsis, little is known about how Nm adapts to permit survival and growth in human blood. In this work we report the transcriptional response of Nm after incubation in human whole blood. The gene expression results indicate that a significant part of the ORFs of the genome (≈30%) is differentially expressed after incubation in human blood, with genes involved in adaptation of Nm metabolism to blood and in virulence and subversion of the host immune system being up-regulated. Combining transcriptional analysis with the generation and characterization of deletion mutants and complementing strains, we identify new factors important for survival in human blood. This first gene expression analysis of Nm in blood significantly increases our knowledge of how this bacterium responds to human blood and causes septicemia. Our results also provide new information on gene function and may ultimately help in the development of diagnostic and therapeutic strategies to control this devastating disease.

## Introduction

*Neisseria meningitidis* (Nm) is a Gram-negative commensal of the human upper respiratory tract and asymptomatic carriage of Nm in the nasopharynx is common in healthy adults. In susceptible individuals, Nm can cause septicemia by crossing the mucosal barrier and entering the bloodstream, or can cause meningitis by crossing the blood–brain barrier and multiplying in the cerebrospinal fluid [1]. Invasive meningococcal infections represent a major childhood disease with a mortality rate of 10% and high morbidity in survivors [2]. During the transition from colonization to an invasive bloodstream infection, Nm must adapt to changing environments and host factors.

Sequencing of different *Neisseria* genomes has facilitated the discovery of many previously unknown virulence factors [3]-[7] and the comparison of disease and carrier strains has recently provided new insights into the evolution of virulence traits in this species [6]. In order to better understand how Nm adapts to different interactions with the host, it is necessary to study the gene expression of the bacterium under conditions that approximate the human niches it encounters *in vivo*. The interactions of Nm with human epithelial and endothelial cells, as well as exposure to human serum, have been analyzed using microarray expression studies, which have provided useful information about the pathogenesis of the bacterium and the function of previously unknown genes, and have also enabled the identification of novel vaccine antigens [8]. However, little is known about how Nm adapts to permit survival and growth in human whole blood, despite the importance of this step in the disease process. An infant rat model of invasive infection has been combined with a signature tagged mutagenesis (STM) approach to identify genes essential for bacteremia [9]. However, Nm is an exclusively human pathogen, and existing animal models may not accurately simulate meningococcal disease. This justifies the use of an experimental system that mimics, as closely as possible, the *in vivo* situation seen during disease. Human whole blood has been used as an *ex vivo* model of sepsis for studying the pathogenesis of Nm in terms of complement activation, cytokine production and immunity [10]–[14]. Similar *ex vivo* models have also been used to understand how pathogens, including *Candida albicans*, *Listeria monocytogenes*, group A and group B *Streptococcus* species, regulate gene expression during exposure to human blood [15]–[18].

In this study we have analyzed the global changes in the transcriptional profile of a virulent Nm serogroup B (NmB) strain in an *ex vivo* model of bacteremia, using incubation in human whole blood and a time-course oligo-microarray experiment. This approach revealed mechanisms used by Nm to adapt to human blood, and was instrumental in analyzing the role of previously known and newly identified virulence factors whose expression was up-regulated during *ex vivo* infection.

## Results and Discussion

### Transcriptome analysis of Nm gene expression in an ex vivo human whole blood model

**Figure 1:**
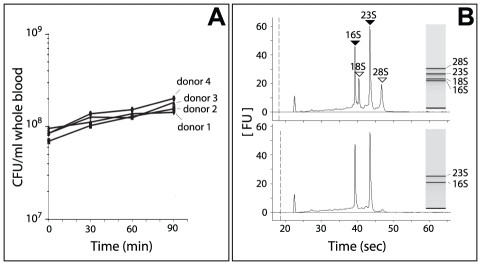
Growth of Nm in human whole blood and RNA analysis. (**A**) Number of bacteria during incubation with human blood. The CFU/ml per single donor is shown during a time course experiment. (**B**) Analysis of isolated total RNA and enriched Nm RNA using a BioAnalyzer 2100 (Agilent). **Upper panel:** Total RNA collected from Nm incubated in human whole blood, bacterial RNA (shaded arrowheads) and eukaryotic RNA (open arrowheads) are indicated. **Lower panel:** Enriched bacterial RNA.

In order to evaluate the transcriptional response of Nm during growth in blood we used an *ex vivo* human whole blood model, which enabled meningococcal responses to both host cellular and humoral bactericidal mechanisms to be analyzed. This *ex vivo* model has shown potential to examine a number of parameters that are likely to be important in the cascade of events associated with acute systemic meningococcal infection [19] and to characterize Nm factors involved in the survival of the bacterium during infection [20], [21]. Freshly isolated whole venous blood collected from four healthy human volunteers (two male and two female) was used. Bacterial loads in patients with fulminant disease can reach up to 10^9^ bacteria/ml [22]–[24]. Therefore, Nm MC58 bacteria (approximately 10^8^, grown in GC medium to early exponential phase) were mixed with blood from each donor in order to mimic disease. Analysis of growth in the blood by colony forming unit (CFU) counting showed that bacterial numbers increased approximately 2-fold over a 90-minute incubation period and that there was no significant difference in the number of CFU between the four donors (Figure 1A).

**Figure 2:**
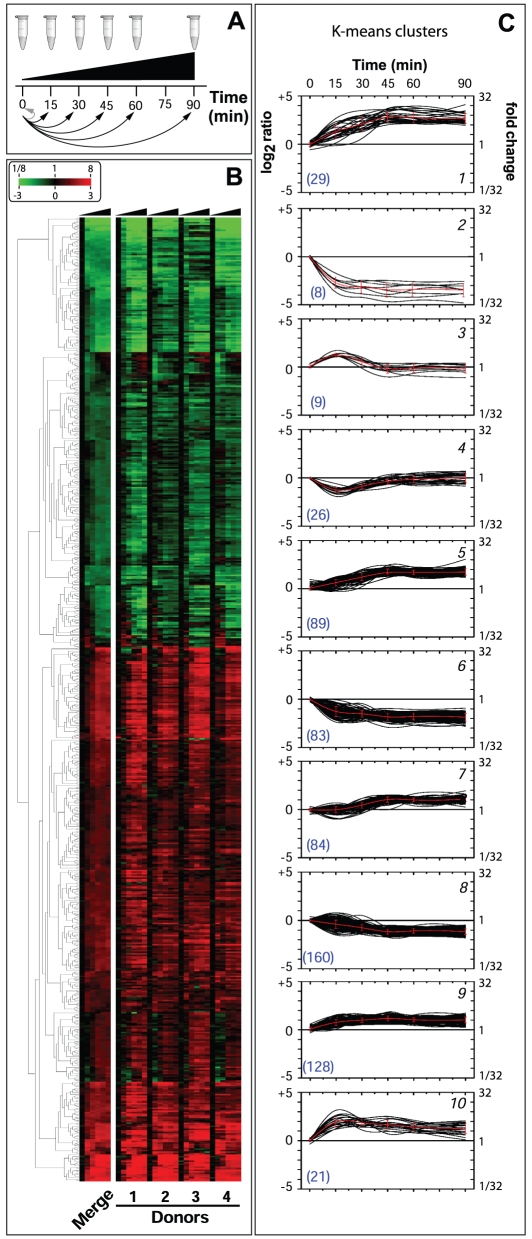
Global changes of Nm gene expression in human whole blood. (**A**) Experimental design. Human blood isolated from four different donors was incubated with Nm and RNA extracted at the indicated time points (samples from each time point was done in triplicate and then pooled). Time 0 was used as the reference time point. (**B**) Hierarchical clustering of the differentially expressed genes showing the data of the four different donors (Donors 1–4) and the average dataset (Merge). Clustering showed two well defined partitions of the expression profiles, 360 up-regulated (red) and 277 down-regulated genes (green). Genes were selected based on a fold change of at least two (log_2_ ratio <−1 or >1) and a t-test *p-value* <0.05. (**C**) Clusters of differentially expressed genes defined by the K-means algorithm and grouped based on the dynamics of expression changes during the time course (black lines) and mean expression values of genes located in defined clusters (red lines). The number of genes included within each cluster is reported in blue between brackets. TIGRFAM main roles and KEGG pathways that significantly correlated with clusters are reported in Table S2.

In order to evaluate the adaptation of Nm to human blood, samples were collected at six different time points (each time point consisted of triplicate cultures): immediately after mixing bacteria with blood (time 0, reference point), and after 15, 30, 45, 60 and 90 minutes incubation at 37°C. Total RNA extracted at each time point consisted of a mix of eukaryotic and prokaryotic RNA (Figure 1B). Since eukaryotic RNA can compete with bacterial RNA during cDNA synthesis and fluorochrome labeling, we used a procedure that simultaneously removes mammalian rRNA and mRNA [25]–[27]. With this procedure, we were able to significantly enrich the samples for Nm prokaryotic RNA (Figure 1B). We then applied an *in vitro* transcription amplification/labeling step [28] to produce amplified-labeled cRNA that was then used in competitive hybridization experiments with a 60-mer Nm oligo-microarray. Transcriptional changes throughout the course of Nm incubation in human blood were defined by comparison of expression levels at various time points against time 0 (Figure 2A). Variability between the four blood donors was quantified by measuring the Pearson correlation coefficient ‘*r’* between the expression matrices of each pair of donors (*r* coefficients between pairs of donor samples (*i,j* = 1-4): *r_1-2_* = 0.77, *r_1-3_* = 0.78, *r_1-4_* = 0.79, *r_2-3_* = 0.74, *r_2-4_* = 0.87, *r_3-4_* = 0.70). We also evaluated the Pearson correlation ‘*r_ijg_*’ at the level of each single gene ‘*g’* and we represented the distribution of the coefficients both globally and between pairs of donors (Figure S1). This analysis showed an excellent agreement between the gene expression profiles obtained from the four donors. The four data sets were averaged in order to obtain a single data set that was subsequently used to evaluate global gene expression changes (Figure 2B).

### Global changes in the Nm transcriptome during growth in human blood

Analysis of the transcriptional profile of Nm grown in human blood from four different donors over a 90 minute time course revealed that a total of 637 genes were differentially regulated during infection, which represents about 30% of the ORFs in Nm genome. Genes were considered to be differentially regulated if they showed, within donor replicas, an average transcript value of log_2_ ratio greater than 1 or less than −1 with a Student's t-test *p-value* lower than 0.05 in at least one time point of the time-course infection, with respect to time 0. False discovery rate estimation was performed by calculating the *q-values* corresponding to a threshold of t-test *p-value* of 0.05, and a range between 0.148 at time 15 min to 0.053 at time 90 min. The consequent number of false positive calls, using the |log_2_(ratio)|>1 cut-off, is relatively stable in each time point and varies between 24 and 28 genes. The selection criterion was also compared with the results obtained with BETR statistics, which is specifically suitable to discover regulated genes during a time course. The BETR algorithm confirmed 509/637 genes as significantly (*p-value* <0.05) regulated genes during the time course. Interestingly, the subset of 128 genes, that are called as possible false positives using the first statistical method, consist of genes strongly regulated with rapidly changing behaviour over time or with blood donor specificity. For this reason we still considered this subset as interesting differentially expressed genes.

The expression profiles of the 637 selected genes were divided into two well-separated groups by hierarchical clustering applied to the expression matrix, with 360 genes up-regulated and 277 genes down-regulated compared to the reference time 0 (Figure 2B). Clusters of co-regulated genes were identified and investigated by performing a Figure Of Merit (FOM) [29] analysis using different clustering algorithms (see Methods). FOM analysis showed that the value was stabilized after a partitioning into 7–10 clusters using all of the algorithms, but with particular quality using the K-means method (FOM_K-means_ is 4.4% to 22.2% less than the FOM of the other clustering algorithms, data not shown). Therefore, the expression profiles were split into 10 clusters according to the K-means partitioning, each of which showed particular expression profile dynamics (Figure 2C). For example, clusters 1, 9 and 10 showed a rapid increase in expression within 15 minutes, after which time gene profiles reached a stable up-regulation. Analogously, clusters 2 and 6 reached a stable down-regulation within 15 minutes. Three additional clusters (5, 7 and 8) reached a stable regulation after a delay of 30 minutes. However, clusters 3 and 4 showed a different dynamic, genes showed up-regulation (cluster 3) or down-regulation (cluster 4) at 15 minutes, but expression levels were restored to the initial relative levels by 30–45 minutes.

**Figure 3:**
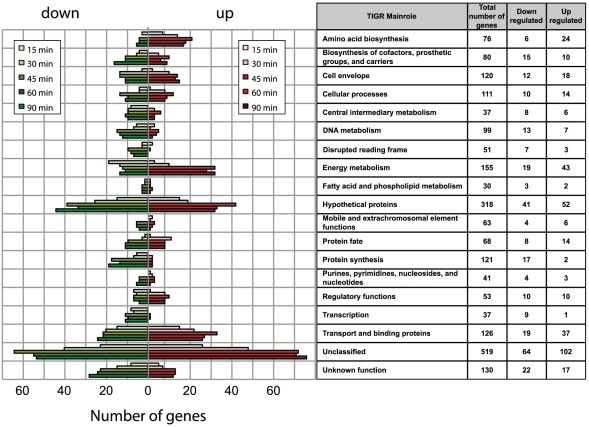
Time course distribution of up- and down- regulated genes within TIGRFAM main roles. The plot reflects the dynamics of Nm metabolic adaptation to blood, and the number of regulated genes within each TIGR family is shown for each time point. The total number of genes in each class and the number of up- and down-regulated genes are listed in the table.

Interestingly, gene expression profiles clustered by K-means partitioning were modularly organized with respect to TIGRFAM functional classes [30] or KEGG metabolic pathways and showed a complete non-overlapping distribution (Table S2). These results suggest that K-means clustering groups genes that are functionally related, which could help in defining the function of un-annotated genes. The genes present in each cluster and the TIGRFAM and KEGG correlation results are reported in Table S1 and Table 2S, respectively. The dynamics of gene expression within each functional class was investigated by plotting the number of regulated genes at each time point for each TIGRFAM class (Figure 3). A wide range of hypothetical, unclassified ORFs and ORFs with unknown function were differentially regulated. Previous transcriptome analysis of Nm grown under various conditions has aided in the functional characterization of unclassified ORFs, including roles in cell adhesion [31] and resistance to antimicrobial peptides [32]. Analysis of the unclassified ORFs regulated in blood may aid in their functional characterization. The major groups of differentially regulated genes are involved in energy metabolism, transport and binding, amino acid biosynthesis, regulatory functions, cellular processes and cell envelope synthesis. These groups are predominantly up-regulated, suggesting a high degree of metabolic adaptation occurs in blood, enabling uptake of different substrates and induction of alternative metabolic pathways. The differential distribution of the number of up- and down- regulated genes within each TIGRFAM class in the initial stages of the time course may indicate the main roles involved in the adaptation process. Intriguingly, the equal distribution of up- and down- regulated genes at 60 and 90 minutes in the majority of functional classes may suggest the initial establishment of equilibrium in the gene expression of Nm physiology.

In order to evaluate if the regulated genes identified are as a result of growth phase changes and not growth in blood per se, we performed microarray analysis of strain MC58 grown in laboratory medium (GC liquid broth) at different time points matching the ones used for analysis in blood (0, 30, 60 and 90 minutes). The growth rate of MC58 (measured as CFU/ml) was comparable between blood and GC (data not shown). The comparison of the dataset generated in GC with the one generated in blood showed that a subset of the differentially regulated genes (≈ 30%) are in common between the two experimental conditions (Table S1). A detailed analysis of these genes showed that they do not correlate with any particular functional class (data not shown). However, despite the fact that some regulated genes are in common and might result from growth phase changes, we decided to include all genes in the subsequent analysis because their altered expression in blood indicates that they are involved in growth and fitness of the meningococcus in this environment. To validate the results obtained in the microarray experiments in blood, quantitative real time PCR (qRT-PCR) was used to analyze the relative expression levels of nine genes from different functional categories (*NMB1030, NMB1870, NMB2091, NMB2132, NMB1567, NMB1541, NMB0995, NMB1946* and *NMB1898*). Experiments were conducted using four biological replicates (each comprising three technical repeats) comparing time 0 versus 45 minutes, using 16S rRNA for normalization. The comparison of gene expression at 45 minutes measured by qRT-PCR and microarrays analyses showed a significant Pearson correlation between the two approaches (*p-value* <0.01, *r* = 0.98; Figure S2B).

### Several regulators are involved in Nm adaptation to human blood

**Figure 4:**
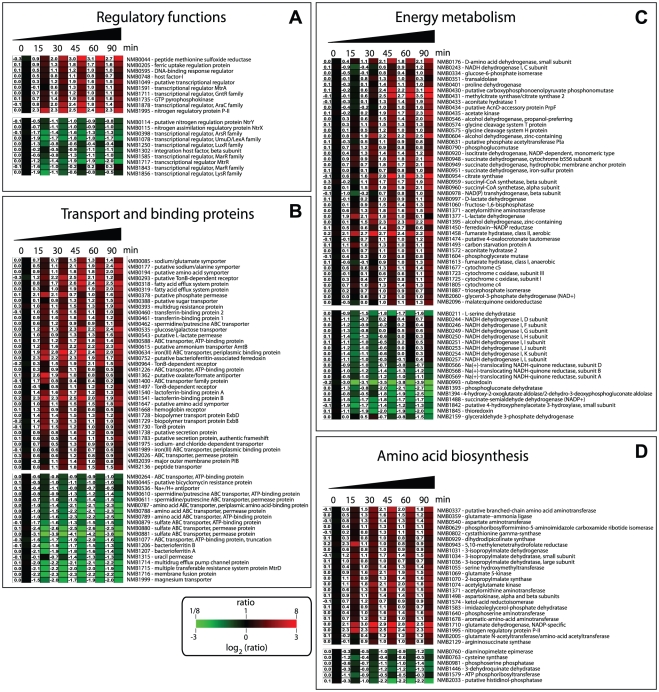
Transcriptional profile of differentially regulated genes grouped by functional TIGRFAM family main roles. Detailed expression profiles of functionally related genes during the time course of Nm in human whole blood. Clusters were created using TMEV. (**A**) Regulatory functions (**B**) Transport and binding proteins (**C**) Energy metabolism (**D**) Amino acid biosynthesis. Each gene is represented by a single row and each time point by a single column; gene identification numbers (based on the MC58 annotation) and gene definitions are reported on the right. Gene expression is displayed in fold change represented by the color bar under the figure. The numerical gene expression values are shown for all the genes at the different time points. For a more detailed analysis, see Table S1.

The expression of numerous regulators was altered during incubation of Nm in human blood (Figure 4A). The ferric-uptake regulation protein (*fur, NMB0205*), which is involved in the regulation of Nm gene expression in response to iron concentration, was significantly up-regulated. Human blood, as well as other body fluids, contains virtually no free iron because extracellular iron is linked to high-affinity iron-binding proteins. The up-regulation of Fur is indicative of iron-limitation, and led to altered expression of several genes in the Fur regulon. In fact, half of the 83 genes regulated by Fur [33], [34], were also differentially regulated in blood with the same pattern of expression seen under iron limitation and/or inactivation of Fur (data not shown).

Hfq (*NMB0748*), a RNA chaperone and key modulator of riboregulation in bacteria, was also up-regulated during incubation in blood. The up-regulation of Hfq in blood suggests that non-coding RNAs might also play a role in Nm infection as recently reported for other bacterial pathogens [18], [35]. Interestingly, the Nm Hfq is involved in stress responses and virulence, with a *hfq* mutant being less able to survive in human whole blood [20] and attenuated in an infant rat model of bacteremia [9]. Further analysis, using a similar *ex vivo* model and a tiling microarray, will be instrumental to identify new Nm non-coding RNAs differently expressed in human blood.

Several other transcriptional regulators were also differentially regulated (Figure 4A), highlighting the high degree of regulation that is required for adaptation of Nm to exposure and survival in blood. In our study we did not observe regulation of the *fnr* gene (*NMB0380*) coding for the Fumarate and Nitrate reductase regulator protein, which is the major player in the metabolic switch from aerobic to anaerobic growth and whose role in Nm infection has been established [36]. However we observed that six FNR-regulated genes were up-regulated (*NMB0388, NMB1805, NMB0577, NMB1677, NMB1623, NMB1870*). This suggests that while the level of expression of *fnr* does not change, the proportion of active FNR is altered during growth in blood. In fact, human blood is an oxygen-restricted environment, due to sequestration of oxygen by hemoglobin, and FNR is expected to be in its active dimerised form leading to increased expression of the FNR regulon including AniA (NMB1623), a nitrite reductase that plays a key role in anaerobic respiration [37].

Two-component regulatory systems (TCS) are one of the most common bacterial signal transduction mechanisms controlling responses and adaptation to environmental changes, and Nm has four predicted TCS [4], [7]: *NMB0114/NMB0115, NMB0595/NMB0594, NMB1249/NMB1250 and NMB1606/NMB1607*. The *NMB0595* gene, which is part of the PhoQ, MisS/PhoP, MisR TCS that has been extensively studied in Nm, was up-regulated throughout the 90 minute time course, but particularly after 30 minutes The partner gene, *NMB0594* coding for the sensor histidine kinase, was slightly up-regulated at 15 minutes but not at later time points. A meningococcal deletion mutant in the *NMB0595* gene displayed an attenuated virulence phenotype in a mouse model of infection [38]. Moreover this TCS has been shown to constitute a functional signal transduction system [39] that modulates the meningococcal virulence factor, lipopolysaccharide [40], and is required for optimal colonization of endothelial cells [41]. On the other hand, *NMB0114/NMB0115* (homologues of the NtrY/NtrX TCS) and *NMB1250* (part of the TCS that exhibits amino acid sequence similarity with NarQ/NarP) were down-regulated. The TCS *NMB1606/NMB1607* was not differently regulated during the incubation in blood. The fact that components of some TCSs were regulated differently may be due to functional interaction and cross-regulation between the TCSs [42] or may be related to the stability of the phosphorylated states of the sensor or regulator and the consequent expression of the genes [43].

### Adaptation of Nm metabolism to human blood

The expression of a large proportion of genes involved in nutrient transport and metabolic pathways was influenced by incubation of Nm in human blood. This indicates a rapid adaptation of the bacterial metabolism to specific nutrients, or nutrient limitations, present in a complex environment such as human blood. The ability of Nm to acquire iron plays an important role in survival within the host, in terms of its ability to replicate within cells [44] and survive in the bloodstream. Nm has evolved numerous iron acquisition systems that enable it to use transferrin, lactoferrin, hemoglobin and haptoglobin-hemoglobin as iron sources [45] and several Nm mutants lacking these iron uptake systems are attenuated in animal models [9], [46]. In this study, iron uptake systems along with the Fur regulator were found to be significantly up-regulated (Figure 4B). Genes encoding the transferrin binding proteins (*tbpA* and *tbpB*), lactoferrin binding proteins (*lbpA* and *lbpB*) and the hemoglobin receptor (*hmbR*) were up-regulated during the time course, together with the genes encoding for the systems involved in the transport of iron through the periplasm: *fbpA* (*NMB0634*) and *tonB/exbB/exbD* (*NMB1728-NMB1730*) (Figure 4B). Interestingly, strains with mutations in genes encoding for TonB, ExbB and ExbD have an attenuated phenotype in the infant rat model of Nm infection [9]. Genes encoding for the iron storage protein bacterioferritin (*NMB1206/NMB1207*) were down-regulated suggesting the necessity for Nm to utilize iron rather than to store it.

Nm can use lactate and glucose as carbon and energy sources, and both compounds are present in human blood [47]. In our study, genes involved in the uptake of glucose (*gluP*, *NMB0535*) and lactate (*lctP*, *NMB0543*) were significantly up-regulated (Figure 4B). Nm catabolizes lactate at a faster rate than glucose and LctP has been shown to be involved in virulence: a *lctP* mutant has a reduced growth rate in cerebrospinal fluid and was attenuated in a mouse model of infection due to increased sensiftivity to complement-mediated killing [48]. Also in the functional class of ‘transport and binding proteins’ we found the up-regulation of genes *NMB0318*–*NMB0319* that are annotated as fatty acid efflux system proteins and are homologues of the *farAB* system of *N. gonorrhoeae*, which are involved in resistance to antibacterial fatty acids [49]. Interestingly, the other system known to be involved in antimicrobial resistance, *mtrCDE* (*NMB1714/NMB1715*), was down-regulated. This different regulation in expression of the antimicrobial systems may be indicative of their specific roles in particular niches within the host. Genes encoding for sulfate, spermidine/putrescine, amino acid, sodium and magnesium transporters were also down-regulated (Figure 4B).

Nm adapted its energy metabolism during incubation in blood, with genes in both aerobic and anaerobic metabolic pathways being regulated. The classification of the up- and down-regulated genes in TIGRFAM sub-roles gave a clear picture of the pathways involved in this adaptation (Figure 4C and Figure S3). We observed up-regulation of genes encoding enzymes involved in glycolysis (*pgi-1, fbp, pgm, tpiA*) and the citric acid cycle (*pprC, acnA, icd, sdhC, sdhD, sdhB, gltA, sucC, sucD, fumC, acnB, fumB, yojH*). Genes encoding for fermentation enzymes were also up-regulated, including genes in a putative 2-methylcitrate pathway (*NMB0430-NMB0433*), which has been shown to be present only in pathogenic *Neisseria* species [50]. Several genes involved in the biosynthesis and assembly of components of the respiratory chain were also differentially regulated (Figure 4C and Figure S3).

Genes included in the TIGRFAM main role ‘amino acid biosynthesis’ were induced in blood, in particular genes involved in glutamate metabolism, indicating that this amino acid may be important nutrient source for Nm in blood (Figure 4D and Figure S3). Indeed, there is evidence that L-glutamate uptake from the host is critical for Nm infection: *gltT* (ABC-type L-glutamate transporter) is essential for meningococcal survival in infected cells and for the establishment of infection in mice [51]; *gdhA* (glutamate dehydrogenase) was found to be important for Nm survival in STM analysis [9] and is hyper-expressed in Nm invasive isolates [52]. The fact that *gdhA* (*NMB1710*) is strongly up-regulated in human blood confirms the important role played by this enzyme in Nm infection. Additionally, genes involved in pyruvate metabolism, which is part of the protein synthesis pathway, were also up-regulated (Figure 4D and Figure S3).

### Regulation of Nm genes involved in host-pathogen interaction

**Figure 5:**
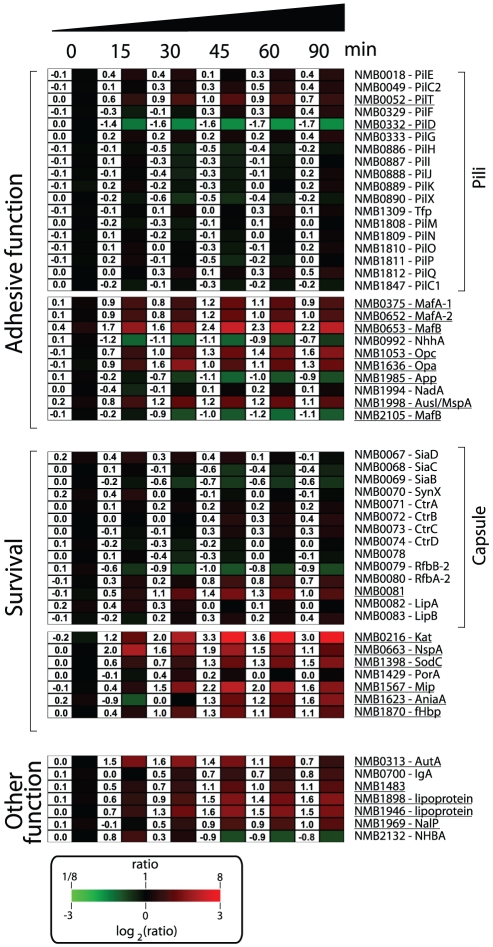
Transcriptional profile of Nm genes coding for proteins involved in host-pathogen interaction. Detailed expression profile of functional genes coding for proteins that play a role in adhesion, survival and other functions. Gene identification numbers (based on the MC58 annotation) and gene names are reported on the right. Genes that were calculated as being significantly regulated are underlined. The numerical gene expression values are shown for all the genes at the different time points.

Nm has evolved to produce an array of molecules to colonize, infect and survive in the hostile microenvironments of the host [1], [53]. A list of genes involved in the mechanisms by which Nm interacts with the host and subverts host defenses is shown in Figure 5. Several genes encoding for molecules with documented or predicted adhesive properties were up-regulated in human blood including, *opa* (*NMB1636*) and o*pc* (*NMB1053*), the gene encoding for AusI/MspA (a phase-variable autotransporter involved in the interaction of Nm with human epithelial and endothelial cells [54], [55]), and the genes encoding for MafA proteins (*NMB0375* and *NMB0652*; homologues of glycolipid-binding adhesins characterized in *N. gonorrhoeae* [56]). Transcription of genes coding for NhhA (*NMB0992*), NadA (*NMB1994*) and App (*NMB1985*), three Nm adhesins involved in interactions with epithelial cells [57]–[59], were not significantly altered during the time course of infection. Also genes coding for pili proteins were not differentially regulated. This might suggest that these factors, which are important for adhesion and colonization, might not be essential during survival in blood.

It is interesting to note that a subset of the genes encoding for surface-exposed adhesins were up-regulated in human blood, suggesting that in addition to their main role in interaction with host tissues, these factors might also be involved in the interaction with blood cells or in the survival in whole blood. For example, Opa and Opc proteins have been reported to have a role in the interaction of Nm to human monocytes [60].

In order to neutralize the effect of reactive oxygen and nitrogen species produced by neutrophils and macrophages, Nm uses enzymes such as catalase, superoxide dismutase and enzymes capable of denitrification [61], [62]. In this study Nm up-regulated the genes coding for catalase (*kat*, *NMB0216*), superoxide dismutase C (*sodC, NMB1398*) and nitrite reductase (*aniA, NMB1623*). Interestingly, SodC has been shown to protect Nm from phagocytosis [63] whereas AniA has also been shown to provide protection to *N. gonorrhoeae* in human sera [64], two important phenotypes in the context of growth in human blood. The *NMB1567* gene was also highly up-regulated in blood, which encodes for a homologue of the *N. gonorrhoeae* Mip (Macrophage Infectivity Potentiator) protein that is involved in intracellular survival and persistence [65].

Nm expresses several surface molecules responsible for effective bacterial defense against human complement. The capsule prevents insertion of the MAC complex into the bacterial outer membrane, while other surface-exposed proteins recruit negative regulators of the complement system such as C4BP and factor H (fH) [66]. Genes involved in capsule biosynthesis were not differentially regulated during the transition from liquid medium to growth in human blood. Similarly, expression of the *porA* gene (*NMB1429*) encoding for the most abundant outer membrane protein of Nm, which is involved in interaction with C4BP [67], was not significantly altered in blood. However, we observed up-regulation of the *fHbp* gene (*NMB1870*), which encodes a surface-exposed lipoprotein able to bind fH and enhance the ability of Nm to multiply and survive within blood [21], [68]. The up-regulation of fHbp in human blood supports previous reports of the crucial role of this protein during Nm pathogenesis. It has been recently shown that the fHbp protein is expressed from two independent transcripts: one bicistronic transcript that includes the upstream gene (*NMB1869*), and a second shorter monocistronic transcript from a FNR-dependent promoter [69]. The upstream *NMB1869* gene was not up-regulated suggesting that the up-regulation of *fHbp* occurs through its own promoter. NspA (Neisseria surface protein A, *NMB0663*) was highly up-regulated throughout the time course of infection. It has been recently reported that NspA is also able to bind fH and enhance resistance to human complement [70]. In this context, up-regulation of the *nspA* gene highlights the important role that this protein is expected to play in survival.

Several genes encoding for other Nm surface-exposed proteins of interest were differently regulated; the gene encoding for autotransporters NalP (*NMB1969*, [71]), IgA protease (*NMB0700*) and AutA (*NMB0313,* [72]) were up-regulated; *NMB1220*, a ligand for the Macrophage Scavenger Receptor A [73], [74] was up-regulated; three putative lipoproteins (*NMB1483*, *NMB1898* and *NMB1946*) were also up-regulated including a NlpD-homologue (*NMB1483*) that has been described as having a role in oxidative damage protection in *N. gonorrhoeae* [75] and in the pathogenesis of *Yersinia pestis* [76].

### Differential expression of genes coding for vaccine antigens against Nm

Prevention of disease caused by Nm serogroups A, C W and Y can effectively be accomplished by vaccination. Recently, several different protein vaccine antigens have been described for Nm serogroup B [77]. This transcriptional analysis has the potential to provide information about the differential regulation of the genes coding for these vaccine antigens in response to human blood and can be predictive for their behavior during *in vivo* infection. The analysis of the dataset showed that several vaccine antigens were differentially regulated, including *NMB0035, tbpA, tbpB, lbpA, lbpB, NMB1030, nspA, opc, NMB1946, NMB1220, NMB2091, fHbp* and *porB* (Table 1). Recently, a Nm serogroup B protein based vaccine (named 4CMenB) has been developed using an *in-silico* genome-based approach [78], [79]. The 4CMenB vaccine contains three main antigens: NadA, fHbp and NHBA. In the vaccine, fHbp was fused with the protein GNA2091 and NHBA was fused with GNA1030. We found that three out of the five genes coding for these antigens were significantly up-regulated in human blood. As already mentioned above, fHbp was up-regulated and also the genes coding for GNA1030 (*NMB1030*) and GNA2091 (*NMB2091*) were highly up-regulated. The other two genes, encoding, for NadA and NHBA, were not differently regulated (Table 1).

**Table 1:** Differential expression of genes encoding for Nm vaccine antigens.

			log_2_					
NMB	Gene	Annotation/Function	15 min	30 min	45 min	60 min		90 min
**Up-regulated genes**								
*NMB0035*		P47, Lipoprotein	1.28	2.82	2.97	2.77		2.09
*NMB0460*	*tbpB*	Transferrin binding protein B	1.04	1.53	1.47	1.13		0.96
*NMB0461*	*tbpA*	Transferrin binding protein A	0.95	1.55	1.81	1.40		1.25
*NMB0663*	*nspA*	Outer membrane protein	2.03	1.61	1.85	1.49		1.13
*NMB1030*		Hypothetical protein	1.06	1.50	1.54	1.44		1.45
*NMB1053*	*opc*	Class 5 outer membrane protein	0.71	1.04	1.28	1.44		1.55
*NMB1220*		Outer membrane protein	0.93	1.49	1.88	1.84		2.05
*NMB1540*	*lbpA*	Lactoferrin binding protein A	1.45	1.65	1.66	1.17		1.27
*NMB1541*	*lbpB*	Lactoferrin binding protein B	2.30	2.33	2.50	1.98		1.89
*NMB1870*	*fHbp*	factor H binding protein	0.39	0.98	1.30	1.12		1.07
*NMB1946*		Outer membrane lipoprotein	0.68	1.31	1.62	1.51		1.46
*NMB2039*	*porB*	Porin B	−0.16	1.14	1.42	0.97		0.80
*NMB2091*		Hypothetical protein	0.78	1.42	1.88	1.69		1.63
***Down-regulated genes***								
*NMB1985*	*app*	Adhesion and penetration protein	−0.24	−0.70	−1.15	−0.98		−0.93
***Not differentially regulated genes***								
*NMB0088*		Transmembrane protein	0.17	0.14	0.15	0.05		0.06
*NMB0928*		Putative lipoprotein	0.09	0.14	0.45	0.38		0.20
*NMB0992*	*nhhA*	Neisseria *hia/hsf* homologue	−1.18^a^	−1.13^a^	−1.15^a^	−0.88		−0.69
*NMB1429*	*porA*	Outer membrane protein PorA	−0.14	0.39	0.18	0.00		0.05
*NMB1988*	*fetA/frpB*	Iron-regulated outer membrane protein	0.86	0.98	0.74	0.04		−0.43
*NMB1994*	*nadA*	Neisseria adhesin A	−0.41	−0.14	0.09	0.18		0.13
*NMB2132*	*nhba*	Neisserial heparin binding antigen	0.82	0.34	−0.90	−0.90		−0.80

a Refers to expression values that do not have a significant p val (<0.05) to be considered differentially regulated.

Transcription of fHbp is increased during oxygen limitation, which may be relevant in an environment like human blood [69] where fHbp plays a protective role against complement activation. NadA is repressed by NadR, but is de-repressed in the presence of 4-hydroxyphenylacetic acid, a metabolite of aromatic amino acid catabolism that is secreted in saliva [80]. As such, NadA may be induced during colonization of the nasopharynx rather than during blood infection. This would also fit with the function of NadA in adhesion to and invasion of the mucosal epithelium [57]. The regulation of NHBA at the molecular level is currently unknown; however the present study suggests that NHBA expression is not influenced by host factors present in human blood. Little is know about the function of GNA1030 and GNA2091. Interestingly, deletion mutants for the two genes display equivalent or slightly reduced levels of survival with respect to the wild-type in both human whole blood and serum [81]. It has also been reported that a GNA2091 deletion mutant is susceptible to membrane stresses, which the meningococcus may encounter in the host during colonization or invasive disease [81].

Localization of proteins within the bacterial cell is an important discriminating factor when they are evaluated as possible antigens for a vaccine development [82]. The *in silico* prediction of localization based on PSORT3 algorithm [83], and available from PSORTdb [84], was combined with blood expression data. In particular, the presence of relevant associations between up and down-regulated groups and protein localization was tested. The PSORT3 algorithm also allowed us to evaluate the presence of specific structures like transmembrane helices and peptide motifs like such as signal peptides. The presence of these structural indicators was evaluated if they were significantly associated with up and down-regulated genes. Interestingly, down-regulated genes are only associated with cytoplasmic membrane proteins, whereas up-regulated genes are associated with proteins located in the periplasmic space or outer membrane (p-value <0.05).

The information regarding the expression of Nm vaccine antigens might give important insight into their role during Nm growth in human blood and might help in their further functional and immunological characterization. Moreover, the dataset of regulated genes that was generated might facilitate the identification of new potential vaccine antigens.

### Identification of genes that contribute to survival of Nm in the ex vivo blood model of infection

**Figure 6:**
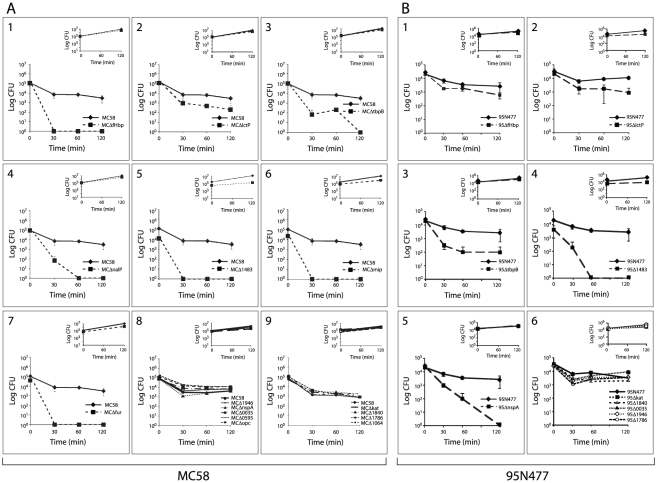
Survival of MC58 and 95N477 wild-type and deletion mutant strains in the *ex vivo* whole blood model of meningococcal septicemia. Deletion mutants in the genetic backgrounds MC58 (panel A) and 95N477 (panel B) of the selected up-regulated genes were tested for survival using the ex vivo whole human blood model over a time course of 120 minutes. The phenotype of the specific mutants were compared to the wild-type strain. MCΔfHbp deletion mutant was used as a control (A1). Deletion mutants with a significant sensitivity to killing by human blood with respect to MC58 wild-type are reported in panels A2-7, while those that were not significantly sensitive are reported in panels A8-9. Deletion mutants with a phenotype in 95N477 genetic background are reported in panels B2-5, while those that were not significantly sensitive to whole human blood are reported in panel B6. Survival of the fHbp deletion mutant in 95N477 is reported in panel B1. The insets of each panel represent the growth control in GC medium for the same time course of incubation as performed in whole blood.

We hypothesized that genes with enhanced expression (up-regulated) in response to incubation in human blood might contribute to the survival of Nm. We selected 15 genes with a significant and sustained increase in expression throughout the time course of blood infection, encoding for proteins belonging to different functional categories: transporters (*tbpB*, *lctP*), host-pathogen interaction (*opc*, *mip, kat, nspA*), surface-exposed proteins (*nalP, NMB1483*), transcriptional regulators (*fur*, *NMB0595*) and hypothetical proteins (*NMB1946*, *NMB0035, NMB1840, NMB1786, NMB1064*) (Table 2). Deletion mutants of each single gene were generated in the Nm strain MC58 by replacing the entire encoding sequence with an erythromycin or kanamycin resistance cassette. The MC58 wild-type and mutant strains were then incubated in human whole blood for two hours and samples were taken at various time points to assess survival through CFU determination. The fHbp mutant strain was used as a control (Figure 6A), since it has previously been described as a crucial factor for survival in human blood [21].

**Table 2:** Nm genes up-regulated in human blood and encoding known and putative virulence factors.

			log_2_ ratio				
NMB	Gene	Annotation/Function	15 min	30 min	45 min	60 min	90 min
*NMB0035*		P47, Lipoprotein	1.28	2.82	2.98	2.77	2.09
*NMB0205*	*fur*	Ferric uptake regulatory protein	0.88	1.33	1.77	1.69	1.57
*NMB0216*	*kat*	Catalase	1.21	2.04	3.33	3.56	2.96
*NMB0460*	*tbpB*	Transferrin binding protein B	1.04	1.53	1.47	1.13	0.96
*NMB0543*	*lctP*	L-lactate permease	1.00	0.93	1.34	1.30	1.62
*NMB0595*		DNA-binding response regulator	0.54	1.15	1.21	1.00	0.96
*NMB0663*	*nspA*	*Neisseria* surface protein A	2.03	1.61	1.85	1.49	1.13
*NMB1053*	*opc*	Class 5 outer membrane protein	0.71	1.04	1.28	1.44	1.55
*NMB1064*		Conserved hypothetical protein NUDIX	1.27	1.89	2.53	2.34	2.29
*NMB1483*		Putative lipoprotein	0.46	0.68	1.08	0.95	1.05
*NMB1567*	*mip*	Macrophage infectivity potentiator	0.42	1.51	2.21	2.03	1.60
*NMB1786*		Hypothetical protein	0.96	1.59	2.19	2.07	2.09
*NMB1840*		Conserved hypothetical protein/integral membrane protein	0.83	1.39	2.35	2.27	2.48
*NMB1946*		Outer membrane lipoprotein	0.68	1.31	1.62	1.51	1.46
*NMB1969*	*nalP*	Serine type peptidase	0.07	0.55	0.93	0.91	1.05

Nm MC58 mutant strains lacking LctP, TbpB, NalP, NMB1483, Mip and Fur were sensitive to killing by human whole blood compared to the MC58 wild-type strain (Figure 6A2–7). Bacterial counts for the other mutants were not significantly different compared to the MC58 wild-type strain (Figure 6A8–9), suggesting that these genes are not essential for Nm survival in human blood in this Nm genetic background. Mutant strains were also characterized for their growth in GC broth at 37°C in the same experimental conditions used for incubation in blood. The growth rate for the majority of the mutants was comparable to the wild-type strain (insets in Figure 6A) suggesting that the phenotype observed in blood is not attributable to a growth defect. In addition, to overcome donor variability, we tested the same mutant strains with a second blood donor and comparable results were obtained (Figure S5).

LctP (*NMB0543*) is a lactate permease involved in the uptake of lactate, which is present in blood and is taken up by the bacterium as a carbon energy source and also converted to precursors of capsular and lipopolysaccharide sialic acid [85]. The up-regulation of *lctP* together with the phenotype of decreased survival that was observed for the deletion mutants in this *ex vivo* model (Figure 6A2) as well as in other relevant models [85], confirm the important role that this membrane transporter plays in increasing complement resistance of Nm strains. However, the fact that the *lctP* deletion mutant is not completely killed in human blood even after 120 minutes of incubation might suggest that other carbon sources could be utilized by the bacterium to generate phospho-enol pyruvate that in turn could be used to generate sialic acid [47].

TbpA and TbpB function as the transferrin receptor in Nm [45] and we demonstrate that a TbpB mutant is defective in growth in human blood, suggesting that transferrin-binding is a crucial step for iron-uptake and survival under these conditions (Figure 6A3).

NalP, an autotransporter lipoprotein with serine-protease activity that is involved in the cleavage of Nm surface-exposed proteins [71], was identified as being important for survival in blood (Figure 6A4). Studies published to date have not identified NalP as a factor involved in the survival of Nm in the host and further study will be necessary to understand if NalP plays a direct role in survival of Nm (*i.e.,* maybe by cleaving a component of the innate immune system), or an indirect role (through activity on one of its known surface targets). NMB1483, a putative surface-exposed lipoprotein annotated as a NlpD-homologue, is also involved in survival in blood (Figure 6A5). BLAST searches reveal that NMB1483 might have a metalloprotease activity and homologues of this protein are involved in the pathogenesis of *N. gonorrhoeae* and *Y. pestis* [75], [76]. Interestingly, in *N. gonorrhoeae* the NMB1483-homologue protects against oxidative damage mediated by hydrogen peroxide and against neutrophil-mediated killing [75].

The *mip* gene is highly up-regulated during blood exposure and the results obtained with the *mip* deletion mutant (Figure 6A6) suggest that the protein has a role in survival of Nm in blood. The Mip lipoprotein is involved in the intracellular survival in macrophages of the closely related species *N. gonorrhoeae* [65] and the Nm Mip protein may also facilitate interaction of Nm with macrophages.

The ferric uptake regulator (Fur) mutants of different pathogens (e.g. *Helicobacter pylori, Staphylococcus aureus*, *Listeria monocytogenes* and *Campylobacter jejuni*) are attenuated in animal models of infections [86]. Similarly, our data demonstrate that in Nm Fur plays a major role in the adaptation and survival of the bacterium in the *ex vivo* model of blood infection (Figure 6A7).

As a next step in investigating the role of the up-regulated proteins in survival in blood, we analyzed a subset of these proteins in another genetic background. We selected the wild-type strain 95N477, where fHbp is expressed at low levels (data no shown) in order to investigate whether other factors that contribute to blood survival are revealed in this strain. We generated 95N477 deletion mutants in the genes encoding for fHbp, LctP, TbpB, NMB1483, NspA, Kat, NMB1840, NMB1946, NMB1786 and NMB0035, which were then tested for survival in human whole blood (Figure 6B). In strain 95N477, deletion of the *fHbp* gene did not significantly alter bacterial survival in blood. However, deletion of NspA in strain 95N477 had a marked effect on survival in blood, suggesting that NspA may be the main factor involved in fH binding and resistance to the alternative complement pathway in this genetic background where fHbp is expressed at very low levels. We did not examine the expression of *fHbp* in 95N477 in blood, however, recent work by Oriente et al. showed that fHbp is regulated in a similar manner in 16/17 strains, in an FNR dependent manner, even in strains with very low fHbp expression [69]. The NspA-related phenotype observed confirms what was recently shown in NspA mutants of a similar Nm strain with low fHbp expression [70] and suggests that the two factors might have a complementary function. The survival phenotypes obtained for LctP, TbpB and NMB1483 mutants were comparable in the two genetic backgrounds suggesting a conserved role of the function of these proteins in the survival of Nm in blood. The other 5 mutants analyzed in 95N477 (*NMB1840*, *NMB1946*, *NMB1786*, *kat* and *NMB0035*) were not sensitive to killing by human blood and showed comparable survivals with respect to MC58 mutants. This suggests that, despite up-regulation of the genes, these factors are not essential for Nm survival in human blood.

**Figure 7:**
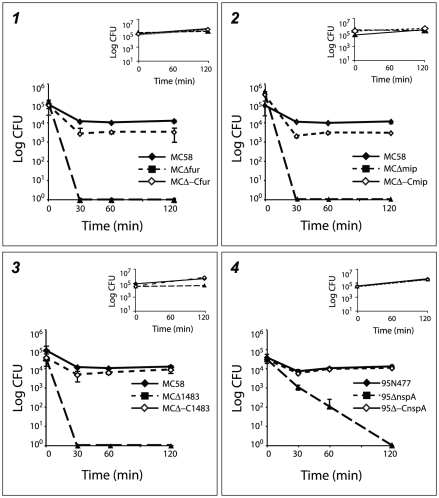
Survival of MC58 and 95N477 complementing strains in the *ex vivo* whole blood model of meningococcal septicemia. Results show the survival of the wild-type, deletion mutant and complementing strains in human whole blood for *fur* (panel 1), *mip* (panel 2) and *NMB1483* (panel 3) in MC58 and *nspA* (panel 4) in 95N477. The insets of each panel represent the growth control in GC medium for the same time course of incubation as performed in whole blood.

In order to better characterize mutant strains with a defective phenotype in blood, deletion mutants for *fur*, *mip and NMB1483* in the MC58 genetic background and *nspA* in the 95N477 genetic background were complemented. The corresponding wild-type gene was inserted as a single copy under the control of a constitutive promoter in the chromosome of the mutant strains generating MCΔ-Cfur, MCΔ-Cmip, MCΔ-C1483 and 95Δ-CnspA. Wild-type, mutant and complementing strains, were tested for growth in whole blood and in rich media as a control. As shown in Figure 7, the survival in blood was restored in all the complementing strains confirming the role played by these factors in the survival of Nm in human blood.

Overall the mutagenesis analysis revealed a role for a subset of the up-regulated genes, showing that a transcriptional approach can be useful for the identification of virulence factors involved in Nm blood survival. However, it is interesting to note that the phenotypes of the mutants do not necessarily correlate with the level of up-regulation of the genes considered. For example, the *kat* gene had the highest level of up-regulation during the time-course but the deletion mutants did not shown any phenotype in the *ex vivo* model. Further study will be necessary to understand the molecular mechanisms behind the phenotypes observed for the deletion mutants analyzed in this study.

### Concluding remarks

**Figure 8:**
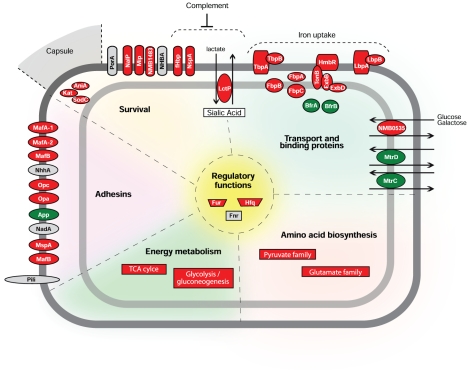
Schematic overview of the factors involved in adaptation and survival of Nm in human blood. The model presents the transcriptional response of Nm genes according to their functional classes (TIGRFAM main roles: energy metabolism, amino acid biosynthesis, transport and binding proteins and regulatory functions) and genes coding for factors involved in host-pathogen interactions (adhesins, survival). The pathways and specific genes mentioned in the results and discussion section are reported. Colors indicate up-regulated genes (red), down-regulated genes (green) and not differentially regulated genes (grey).

Characterization of the bacterial transcriptome during host-pathogen interactions is a fundamental step for understanding infectious processes caused by human pathogens. Some steps of Nm-host interactions have been analyzed by microarray expression profiling [8]. However, despite its importance in the disease process, little is known about how Nm adapts to permit survival and growth in blood. In this work, we characterized the transcriptional profile of a Nm strain in an *ex vivo* human whole blood model of infection for the first time, showing how this bacterium adapts to enable survival and growth in blood. Nm undergoes a rapid, adaptive response and, as a consequence, bacterial metabolism and virulence pathways are remodeled resulting in enhanced survival, which could enable bacterial dissemination and proliferation. A graphical summary of our findings is shown in Figure 8, where we report a model representing the transcriptional response of Nm genes according to their functional class (energy metabolism, amino acid biosynthesis, transport and binding proteins) and role in host-pathogen interactions (adhesins, survival). We suggest that a complex regulatory network controls the changes in expression seen upon exposure to blood. The transcriptional and functional studies reported in this work establish that Fur represents a major regulator of adaptation to human blood, and that the genes involved in iron acquisition and storage are one of the main functional classes activated in the adaptation process. Our transcriptional and functional results suggest that Hfq and Fnr are also playing a role in the adaptation to human blood.

Through mutagenesis studies of a subset of up-regulated genes we were able to confirm the role of previously known virulence factors in this *ex vivo* model and to identify new virulence factors based on their importance for bacterial survival in human blood.

Nm usually lives as a commensal bacterium in the upper airways of humans. Occasionally some strains can cause life-threatening diseases such as sepsis and bacterial meningitis. It has been proposed that differences in the pathogenic potential between carriage and disease isolates might be influenced by small genetic differences in genes from the core genome [6]. However, differences in the pathogenic potential of strains can be also attributed to differences at the transcriptional level that can be identified by performing comparative transcriptomic analyses of different Nm strains under conditions that approximate the human niches the bacterium encounters *in vivo*. Joseph *et al*. used this approach to compare the transcriptional responses of two NmB strains using a nasopharyngeal cell model of adhesion [87]. We propose that the analysis of the transcriptional response of Nm strains using the *ex vivo* model of blood infection might represent a new approach to discriminate between the virulence potentials of different isolates.

In the field of Nm pathogenesis, this first analysis of gene expression in blood significantly increases the knowledge of how this bacterium responds to human blood and causes sepsis. The findings reported in this study could also be helpful to identify the function of gene products annotated as hypothetical proteins, understand the regulation of vaccine antigens in blood and ultimately develop diagnostic and therapeutic strategies to control a devastating disease.

## Materials and Methods

### Ethics statement

The institutional review board of the department of health service at Novartis Vaccines and Diagnostics (Siena, Italy) approved the study and the use of human blood from the four volunteers. Written, informed consent was obtained from the adult participants.

### Bacterial strains and growth conditions

Serogroup B Nm strains (MC58, 95N477 and their isogenic derivatives) and *Escherichia coli* DH5α strains used in this study are listed in Table S5. Nm strains were grown on GC agar plates or in GC broth at 37°C in 5% CO_2_. *E. coli* strains were cultured in Luria-Bertani (LB) agar or LB broth at 37°C. Antibiotics were added when required; erythromycin and chloramphenicol were added at final concentration of 5 µg/ml for selection of deletion mutants and complementing strains, respectively. Ampicillin and erythromycin were added at final concentration of 100 µg/ml for *E. coli*.

For microarray analysis of Nm in whole blood, MC58 Nm strains were grown overnight on GC agar plates and cultured in GC broth to early exponential phase. Approximately 10^8^ bacteria were pelleted by centrifugation at 4000 rpm for 5 minutes and resuspended in an equal volume (1 ml) of freshly isolated human blood maintained at 37°C. Whole blood infected with bacteria was incubated for 0, 15, 30, 45, 60 and 90 minutes with gentle shacking to avoid sedimentation. Samples were then treated with RNA protect bacteria reagent (Qiagen) immediately after adding bacteria to whole blood (time 0) and for each time point to 90 minutes. Cells were harvested by centrifugation and stored at −80°C until bacterial RNA isolation. Each time point was represented by three samples from which RNA was purified separately. CFU counts were obtained for Nm cultures immediately before time course initiation (time 0) and after 30, 60 and 90 minutes of incubation. The time course experiments were performed independently with four different blood donors.

For microarray analysis of Nm in GC broth medium, MC58 strain was grown and treated as described above for microarray analysis in whole blood. Briefly, approximately 10^8^ bacteria were resuspended in an equal volume (1 ml) of GC broth medium containing heparin at final concentration of 5 U/ml. Bacteria were incubated for 0, 30, 60 and 90 minutes. Samples were then treated as described above. Each time point was represented by three samples from which RNA was purified separately. CFU counts were obtained, immediately before time course experiment (time 0) and at 30, 60 and 90 minutes. This time course experiment was performed in duplicate with two different bacterial growths.

### Human whole blood

Heparinized human venous blood was collected from 4 healthy volunteers (two males and two females, ages ranged from 25 to 35 years). Heparin was used at concentration of 5 U/ml. Heparin was used in preference to ethylenediaminetetraacetic acid (EDTA) as an anti-coagulant agent because EDTA chelates divalent cations, which would influence cellular functions during *Neisseria*-blood cell interactions. Heparin is commonly used in blood models of infection for *Neisseria* [11], [19], and while heparin has been reported to affect complement activation, it is not known to alter gene expression in *Neisseria*.

### Isolation and enrichment of bacterial RNA

For RNA isolation, the samples were incubated with 5 volumes of Erythrocyte Lysis (EL) buffer (Qiagen) for 15 minutes on ice and centrifuged at 4°C and 4500 rpm for 6 minutes. Total RNA (bacterial and eukaryotic RNA) was isolated by enzymatic lysis using lysozyme (Sigma) at 0.4 mg/ml final concentration, vortexed and incubated at room temperature for 5 minutes. RNA isolation was completed with the RNeasy Mini Kit (Qiagen) according to the manufacturer's protocol. DNA contamination was avoided by on-column treatment and post-treatment with RNase-Free DNase Set (Qiagen). Absence of bacterial DNA was confirmed by PCR with primers specific for *NMB0992*. RNA concentration and integrity was assessed by measurement of the A260/A280 ratios and electrophoretic analysis with an Agilent 2100 Bioanalyzer (Agilent Technologies).

Finally, three total RNA aliquots corresponding to each time point were pooled and used for the bacterial RNA enrichment procedure. Enrichment of bacterial RNA was performed using the MICROBEnrich kit (Ambion) according to manufacturer's instructions. The percentage of enrichment of bacterial RNA from the initial total RNA mixture (eukaryotic and bacterial RNA) was reproducible between the different independent experiments performed (single donors) and was usually around 10%. Enriched-bacterial RNA was assessed by electrophoretic analysis with an Agilent 2100 Bioanalyzer. The absence of eukaryotic nucleic acids was confirmed by PCR and RT-PCR with primers specific for the *actB* human gene. Enriched-bacterial total RNA was used as template for cRNA synthesis (amplification and labeling reaction) and also for validation of microarray data by qRT-PCR.

### RNA amplification and labeling

Enriched-bacterial RNA was amplified and labeled using the MessageAmp II Bacteria kit (Ambion). The kit employs an *in vitro* transcription (IVT)-mediated linear amplification system to produce amplified RNA (cRNA). Briefly, 100 ng of total RNA from each time point was used as template for the synthesis reaction. An initial polyadenylation step was performed. The tailed RNA was reverse transcribed (cDNA synthesis) in a reaction primed with oligo (dT) primers bearing a T7 promoter. The resulting cDNA was then transcribed with T7 RNA Polymerase to generate antisense RNA (cRNA) in an *in vitro* reaction for 14 hours at 37°C. The cRNA was labeled by including Cyanine 3-CTP (Cy3) and Cyanine 5-CTP (Cy5; Perkin Elmer, Boston, MA) nucleotides in the IVT reaction.

The cRNA was then fragmented in fragmentation buffer (Agilent Technologies) at 60°C for 30 minutes before hybridization. Competitive hybridizations were conducted with 500 ng of Cy3-labelled cRNA reference (bacteria in contact with whole blood at time 0) versus 500 ng Cy5-labelled cRNA of each time point (15, 30, 45, 60 and 90 minutes). cRNAs were hybridized onto the microarray slides for 17 hours at 65°C, washed and scanned with an Agilent scanner following the Agilent Microarray protocol.

### Microarray design and analysis

Gene expression analysis was performed using an Agilent custom-designed 60-mer oligonucleotide microarray. Probes were designed to cover the complete annotated genes of Nm MC58 strain, except for 80 genes that for cross-homology reasons it was not possible to design specific probes and the final resulting coverage is 96.2% (2078/2158 genes). Probe design was performed respecting oligonucleotide sequence specificity, structural and thermodynamic constraints [88], [89] in order to obtain at least two probes for each open reading frame of the genome. For some specific genes we covered in tiling the entire open reading frames (*fHbp*, *nadA*, *nhba, NMB1030, NMB2091*). Two probes for each of the previous selected genes together with the seven multi locus typing housekeeping genes, *abcZ*, *adk*, *aroE*, *fumC*, *gdh*, *pdhC* and *pgm* were used for the design of 21 and 5 replicas randomly distributed on the surface of the chip to have control spots of the signal uniformity within each sample. The chip layout was submitted to the EBI ArrayExpress and it is available with the identifier A-MEXP-1957.

Normalization was performed with the application BASE [90] using an intra-slide median centering after a low intensity spot correction (if the average spot intensity, background subtracted, is less than one standard deviation of the background signal the intensity spot was corrected to the same value of one standard deviation of the background signal).

Differentially expressed genes were assessed by grouping, at each time point independently, all log_2_ ratio values corresponding to each gene, within experimental replicas and spot replicas, and comparing them against the zero value by Student's t-test statistics (one tail). We usually accept as differentially expressed those genes having a t-test *p-value* <0.05 in at least one time point. We also applied a log_2_ ratio threshold filtering in the same time point, accepting values >1 or <−1. The threshold is inferred from log_2_ ratio distributions widths (standard deviation between 0.77 and 1.60 and an average of 1.08) observed in each sample at time points from 15 to 90 minutes. Multiple testing problem and Type I error rate control was done by false discovery rate (FDR) statistics based on the estimation of the *q-values* for each t-test [91].The previous criterion to define differentially expressed genes was compared with the results obtained with BETR [92], a Bayesian statistical method specifically developed to assess differentially expressed genes during a time course experiment.

### Clustering and enrichment analysis

The Figure Of Merit (FOM) [29] analysis is a quantitative method to establish the internal quality of clusters inferred with a defined clustering algorithm. In particular the FOM algorithm assesses the quality of the clustering by a jackknife approach removing one experiment ‘*e’* at a time from the dataset, clustering gene profiles for the remaining experiments and evaluating the root mean square deviation in the left-out condition ‘*e’* of the individual gene expression levels relative to their cluster means. See references [29], [93] for further details. We measured the aggregate adjusted FOM for different clustering algorithms as implemented by the *R* package *clValid* applied to the averaged expression profiles of the 637 genes that we selected as regulated. Hierarchical, Self Organizing Tree Algorithm (SOTA), Partitioning Around Medoids (PAM) and K-means clustering algorithms, applied as described below, were compared and FOM was plotted as a function of the increasing number of clusters. When the number of clusters increases, FOM tends to decrease and stabilize to a plateau. Better clustering is obtained using algorithms that reach this plateau faster.

Hierarchical clustering was applied as implemented by *hclust* in the *R* package *stats* using the Euclidean metrics and the average agglomeration method.

The SOTA was applied as implemented by the same *R* package *clValid*, also based on the Euclidean metrics with unchanged default parameters.

The PAM method was applied as implemented by the *R* package *cluster* with Euclidean metrics and default parameters.

K-means clustering was applied as implemented by the *R* packages *clValid* and *stats* with Euclidean metrics and default parameters.

Enrichment analysis was performed by Fisher's exact test and Benjamini-Hochberg correction as implemented by the MultiExperiment Viewer TMEV [94] testing whether the list of genes up/down regulated or belonging to a specific cluster was particularly rich, with respect to a random distribution, of genes annotated in a specific TIGRFAM term (release 7.0) or belonging to a particular KEGG metabolic pathway (release 48.0).

### qRT-PCR analysis and normalization

The microarray data was validated by two-step qRT-PCR for nine differentially regulated genes for one time point (45 minutes) for each single donor. Primers for genes *NMB0995, NMB1030*, *NMB1541, NMB1567, NMB1870*, *NMB1898, NMB1946, NMB2091* and *NMB2132* are reported in Table S3. They were designed using the Primer3 program and primer specificity was controlled following the denaturing protocol of the MX3000P Real Time PCR system Software version 2.0. Standard curves were performed using genomic DNA from Nm to measure primer pair efficiency. Specificity of all amplicons was confirmed by melting curves and gel analysis. For analysis of *in vitro* transcripts derived from Nm growth in human whole blood, cDNA synthesis was primed using 500 ng of total RNA, random hexamer primers (Promega) and SuperScript II (Invitrogen) according to the manufacturer's instructions.

Fluorescence PCR amplifications were performed using 1 µl aliquot of each first strand cDNA reaction with Brilliant SYBR Green QPCR Master mix (Stratagene) on a Mx3000P cycler (Stratagene) in a final volume of 25 µl. The qRT-PCR reactions were performed in triplicate for the four biological replicates. To normalize the data, 16S rRNA was used as an endogenous control, which is transcribed at constant levels. Relative quantification was performed using the 2^(-ΔΔCt)^ method, including an efficiency correction for the primers using the Relative Expression Software Tool (REST).

### Construction of isogenic deletion mutants and complementing strains

To generate isogenic deletion mutants, target genes were truncated by replacing the gene sequence with an erythromycin (Ery) or kanamycin (Kan) resistance cassette. Approximately 800 bp fragments of the flanking regions of target genes were amplified by PCR from Nm MC58 genomic DNA. Primers used for generation of flanking regions are listed in Table S3. Upstream regions were generated with XbaI and SmaI restriction sites, while downstream regions with SmaI and XhoI restriction sites. Restriction enzymes were purchased from New England Biolabs. The purified PCR fragments were digested and co-ligated into the pBluescript (pBS-KS vector) (Novagen) and transformed into *E. coli* DH5-α using standard techniques. Resulting plasmids were digested with SmaI to insert the erythromycin or kanamycin cassette. All constructs generated to delete the target genes are listed in Table S4. Once subcloning was complete, plasmid DNA was linearized using ApaI and naturally competent Nm MC58 and 95N477 strains were transformed. Transformants were then selected on plates containing erythromycin at 5 µg/ml or kanamycin at 100 µg/ml. Deletion of the target gene was verified by colony PCR analysis using primer pairs to amplify a PCR product inside each gene locus (Table S3). In addition, the primers 1 and 2 (Figure S4A and Table S3) were used to confirm, by sequencing, the correct insertion and orientation of antibiotic resistance cassettes within the deletion region. Furthermore, the sequencing permitted verification that there were no variations in the sequences of the adjacent genes (used as flanking regions) due to the recombination event. The amplified PCR fragments were sequenced using the primers 1 and 2 specific for each gene region and the primers 3 and 4 specific for the antibiotic cassette used to generate the KO strains (Kan or Ery) (Figure S4A and Table S3). Southern blots analysis was performed to verify that all the deletion mutants have no off-target insertions of the antibiotic resistance cassettes.

### The various deletion mutant strains were also analyzed for growth kinetics in GC broth with respect to the wild-type strains

The genes to be complemented were cloned into the pCom-pRBS vector [95]. Forward primers include a NdeI restriction site and reverse primers contain a NsiI restriction site. The pairs of primers used to amplify the different genes are indicated in Table S3. PCRs were performed on Nm MC58 (for genes: *NMB1483* and *mip*) or 95N477 (for gene *nspA*) genomic DNA using the Platinum Taq High Fidelity DNA polymerase (Invitrogen). Each PCR product was digested with NdeI and NsiI enzymes, and cloned into the NdeI/NsiI sites of the pCom-pRBS vector. Resulting plasmids were checked by sequencing and are listed in Table S5. Plasmid DNA was linearized using SpeI and used to transform respective isogenic deletion mutants. The genes were inserted in the intergenic region between the *NMB1428* and *NMB1429* genes and the recombination event occurs between the upstream and downstream region of this locus, allowing the insertion of chloramphenicol resistance cassette and the gene of interest under the control of a constitutive promoter. Transformants were then selected on plates containing chloramphenicol at 5 µg/ml and insertion of the target gene was verified by colony PCR using primers designed on the regions flanking the site of recombination as previously described [95].

The various deletion mutant and complementing strains were also analyzed for growth kinetics in GC broth with respect to the wild-type strains.

### Southern blot analysis

Genomic DNA of Nm wild-type and deletion mutant strains were isolated using Dneasy blood and tissue extraction kit (Qiagen) according to the manufacturer's instructions. Two µg of genomic DNA was digested with BglI overnight and purified using a PCR purification kit (Qiagen). Southern Blot was performed using the ECL Direct Nucleic Acid Labeling and Detection Systems (GE Healthcare) according to the manufacturer's instructions. Briefly, digested DNA was separated in a 0.8% agarose gel and blotted to Hybond N+ membrane (Amersham). The blots were hybridized with the probes generated by PCR using High Fidelity DNA Polymerase (Invitrogen) and labeled with HRP (horseradish peroxidase). The probes were a 1019 bp-Kan (amplified using the primers SB_Kan Fw and SB_Kan Rv) or a 1018 bp Ery fragment (amplified using the primers SB_Ery Fw and SB_Ery Rv) (Table S3).

### Ex vivo whole blood model of meningococcal bacteremia

Nm MC58 and 95N477 wild-type, deletion mutants and complementing strains were grown on GC agar plates at 37°C overnight. Bacteria were harvested into GC liquid medium to an OD = 600 nm of 0.05 and grown to mid-log phase (OD 0.5-0.6) then diluted to approximately 10^6^, 10^5^ or 10^4^ CFU/ml in a total volume of 100 µl of GC liquid medium in a 96 well/plate. The assay was started by the addition of 100% whole human blood (190 µl), supplied by two different donors (a male and a female) to the bacterial suspension (10 µl) in a 96 well/plate. Cultures were incubated at 37°C/5%CO_2_ with gentle agitation, at various time points (30, 60 and 120 minutes) an aliquot of the sample was removed and the number of viable bacteria determined by plating serial dilutions onto MH agar and incubating overnight at 37°C/5% CO_2_. Experiments were performed in duplicate. We used the diluted 100 µl culture as the control time 0 (T0). Whole venous blood, collected from healthy individuals and anti-coagulated with heparin (5 U/ml), was used for whole blood experiments [11], [19].

## Supporting Information

Figure S1 Statistical analysis of the correlation between donor samples. (A) Top panel, distribution of Pearson correlation *r_ijg_* of gene expression profiles (*g*) between donor samples *i* and *j* bottom panel, the same distribution results shown as a box-plot. Typically genes show a good reproducibility (3/4 of the comparisons have *r* > 0.6). (B) Same analysis as performed in A but comparing pairs of data sets between the single blood donors shown in single box-plots. (C) Quartile statistics values are reported in the table for pairs of donors shown in panel B.
(TIF)

Figure S2 Validation of microarray data by qRT-PCR. (A) Comparison of microarray (white bars) and qRT-PCR (black bars) fold change results for 9 selected genes. Fold change qRT-PCR ratios represent the difference in transcript abundance/signal for these genes after 45 minutes of incubation in human whole blood as compared to time 0. qRT-PCR normalization data was done using 16S rRNA as a reference gene. The qRT-PCR and microarray data are represented as the mean of gene expression of four independent experiments. (B) Correlation analysis of microarray and qRT-PCR transcript measurements for the nine selected genes shown in panel A. The qRT-PCR and microarray log_2_ values were plotted and the coefficient of correlation was calculated, *r*  =  0.98.
(TIF)

Figure S3 Distribution of differentially regulated genes within TIGRFAM sub-roles and KEGG pathways. (A) TIGR families sub-roles and (B) KEGG pathways. The groups with five or more differentially regulated genes are represented. Darker bars indicate the most relevant enrichments among the included groups (Fisher s exact test *p-value* < 0.05).
(TIF)

Figure S4 Characterization of the Nm deletion mutants. (A) Schematic representation of the allelic replacement with the resistance cassette, used to generate the deletion mutant strains. The gene locus of each deletion mutant was amplified from the genomic DNA using primers specific for each amplicon (indicated as 1 and 2) and the PCR fragments were sequenced using the same primers and primers 3 and 4 for the antibiotic cassette Kan or Ery. (B) The orientation of the resistance cassette for each deletion mutant, determined from sequencing. C. Southern blot analysis was performed using labeled Kan and Ery PCR products as probes. Genomic DNA of wild-type and deletion mutant strains was digested with *BglI*. The size of the expected fragment for each mutant is reported in panel B. The length of the fragment is approximate since the *BglI* restriction site is subjected to *dam* methylation that could occur along the genomic DNA.
(EPS)

Figure S5 Survival of MC58 wild-type and deletion mutant strains in the *ex vivo* whole blood model using a second blood donor. Deletion mutants of the selected up-regulated genes were tested for survival using the *ex vivo* whole human blood model over a time course of 120 minutes. In each panel the phenotype of the specific mutant is compared to MC58 wild-type strain. The MCDfHbp deletion mutant was used as a control. The insets of each panel represent the growth control in GC medium for the same time course of incubation as done with whole blood.
(TIF)

Table S1 List of genes belonging to the different K-means partitioning clusters and TIGRFAMs main roles. Provided as an Excel file.
(XLS)

Table S2 Significant correlation (Fisher s exact test *p-value* < 0.05, underlined if significant with Benjamini-Hochberg correction) between K-means partitioning clusters and TIGRFAM main roles and KEGG pathways. Provided as an Excel file.
(XLS)

Table S3 Primers used in this study.
(DOC)

Table S4 Plasmids used in this study.
(DOC)

Table S5 Nm wild-type, deletion mutants and complementing strains used in this study.
(DOC)

## References

[1] Virji M (2009). Pathogenic neisseriae: surface modulation, pathogenesis and
infection control.. Nat Rev Microbiol.

[2] Stephens DS, Greenwood B, Brandtzaeg P (2007). Epidemic meningitis, meningococcaemia, and Neisseria
meningitidis.. Lancet.

[3] Bentley SD, Vernikos GS, Snyder LA, Churcher C, Arrowsmith C (2007). Meningococcal genetic variation mechanisms viewed through
comparative analysis of serogroup C strain FAM18.. PLoS Genet.

[4] Parkhill J, Achtman M, James KD, Bentley SD, Churcher C (2000). Complete DNA sequence of a serogroup A strain of Neisseria
meningitidis Z2491.. Nature.

[5] Peng J, Yang L, Yang F, Yang J, Yan Y (2008). Characterization of ST-4821 complex, a unique Neisseria
meningitidis clone.. Genomics.

[6] Schoen C, Tettelin H, Parkhill J, Frosch M (2009). Genome flexibility in Neisseria meningitidis.. Vaccine.

[7] Tettelin H, Saunders NJ, Heidelberg J, Jeffries AC, Nelson KE (2000). Complete genome sequence of Neisseria meningitidis serogroup B
strain MC58.. Science.

[8] Claus H, Vogel U, Swiderek H, Frosch M, Schoen C (2007). Microarray analyses of meningococcal genome composition and gene
regulation: a review of the recent literature.. FEMS Microbiol Rev.

[9] Sun YH, Bakshi S, Chalmers R, Tang CM (2000). Functional genomics of Neisseria meningitidis
pathogenesis.. Nat Med.

[10] Hellerud BC, Stenvik J, Espevik T, Lambris JD, Mollnes TE (2008). Stages of meningococcal sepsis simulated in vitro, with emphasis
on complement and Toll-like receptor activation.. Infect Immun.

[11] Ison CA, Heyderman RS, Klein NJ, Peakman M, Levin M (1995). Whole blood model of meningococcal bacteraemia–a method for
exploring host-bacterial interactions.. Microb Pathog.

[12] Nolte O, Rickert A, Ehrhard I, Ledig S, Sonntag HG (2002). A modified ex vivo human whole blood model of infection for
studying the pathogenesis of Neisseria meningitidis during
septicemia.. FEMS Immunol Med Microbiol.

[13] Sprong T, Brandtzaeg P, Fung M, Pharo AM, Hoiby EA (2003). Inhibition of C5a-induced inflammation with preserved
C5b-9-mediated bactericidal activity in a human whole blood model of
meningococcal sepsis.. Blood.

[14] Welsch JA, Granoff D (2007). Immunity to Neisseria meningitidis group B in adults despite lack
of serum bactericidal antibody.. Clin Vaccine Immunol.

[15] Fradin C, Kretschmar M, Nichterlein T, Gaillardin C, d'Enfert C (2003). Stage-specific gene expression of Candida albicans in human
blood.. Mol Microbiol.

[16] Graham MR, Virtaneva K, Porcella SF, Barry WT, Gowen BB (2005). Group A Streptococcus transcriptome dynamics during growth in
human blood reveals bacterial adaptive and survival
strategies.. Am J Pathol.

[17] Mereghetti L, Sitkiewicz I, Green NM, Musser JM (2008). Extensive adaptive changes occur in the transcriptome of
Streptococcus agalactiae (group B streptococcus) in response to incubation
with human blood.. PLoS ONE.

[18] Toledo-Arana A, Dussurget O, Nikitas G, Sesto N, Guet-Revillet H (2009). The Listeria transcriptional landscape from saprophytism to
virulence.. Nature.

[19] Ison C, Pollard AJaM MCJ (2001). Whole-Blood Model.. Meningococcal Vaccines: Methods and Protocols.

[20] Fantappie L, Metruccio MM, Seib KL, Oriente F, Cartocci E (2009). The RNA chaperone Hfq is involved in stress response and
virulence in Neisseria meningitidis and is a pleiotropic regulator of
protein expression.. Infect Immun.

[21] Seib KL, Serruto D, Oriente F, Delany I, Adu-Bobie J (2009). Factor H-binding protein is important for meningococcal survival
in human whole blood and serum and in the presence of the antimicrobial
peptide LL-37.. Infect Immun.

[22] Darton T, Guiver M, Naylor S, Jack DL, Kaczmarski EB (2009). Severity of meningococcal disease associated with genomic
bacterial load.. Clin Infect Dis.

[23] Hackett SJ, Guiver M, Marsh J, Sills JA, Thomson AP (2002). Meningococcal bacterial DNA load at presentation correlates with
disease severity.. Arch Dis Child.

[24] Ovstebo R, Brandtzaeg P, Brusletto B, Haug KB, Lande K (2004). Use of robotized DNA isolation and real-time PCR to quantify and
identify close correlation between levels of Neisseria meningitidis DNA and
lipopolysaccharides in plasma and cerebrospinal fluid from patients with
systemic meningococcal disease.. J Clin Microbiol.

[25] Garzoni C, Francois P, Huyghe A, Couzinet S, Tapparel C (2007). A global view of Staphylococcus aureus whole genome expression
upon internalization in human epithelial cells.. BMC Genomics.

[26] Maurer AP, Mehlitz A, Mollenkopf HJ, Meyer TF (2007). Gene expression profiles of Chlamydophila pneumoniae during the
developmental cycle and iron depletion-mediated persistence.. PLoS Pathog.

[27] Orihuela CJ, Radin JN, Sublett JE, Gao G, Kaushal D (2004). Microarray analysis of pneumococcal gene expression during
invasive disease.. Infect Immun.

[28] Francois P, Garzoni C, Bento M, Schrenzel J (2007). Comparison of amplification methods for transcriptomic analyses
of low abundance prokaryotic RNA sources.. J Microbiol Methods.

[29] Yeung KY, Haynor DR, Ruzzo WL (2001). Validating clustering for gene expression data.. Bioinformatics.

[30] Haft DH, Loftus BJ, Richardson DL, Yang F, Eisen JA (2001). TIGRFAMs: a protein family resource for the functional
identification of proteins.. Nucleic Acids Res.

[31] Grifantini R, Bartolini E, Muzzi A, Draghi M, Frigimelica E (2002). Previously unrecognized vaccine candidates against group B
meningococcus identified by DNA microarrays.. Nat Biotechnol.

[32] Frigimelica E, Bartolini E, Galli G, Grandi G, Grifantini R (2008). Identification of 2 Hypothetical Genes Involved in Neisseria
meningitidis Cathelicidin Resistance.. J Infect Dis.

[33] Delany I, Grifantini R, Bartolini E, Rappuoli R, Scarlato V (2006). Effect of Neisseria meningitidis fur mutations on global control
of gene transcription.. J Bacteriol.

[34] Grifantini R, Sebastian S, Frigimelica E, Draghi M, Bartolini E (2003). Identification of iron-activated and -repressed Fur-dependent
genes by transcriptome analysis of Neisseria meningitidis group
B.. Proc Natl Acad Sci U S A.

[35] Papenfort K, Vogel J (2010). Regulatory RNA in bacterial pathogens.. Cell Host Microbe.

[36] Bartolini E, Frigimelica E, Giovinazzi S, Galli G, Shaik Y (2006). Role of FNR and FNR-regulated, sugar fermentation genes in
Neisseria meningitidis infection.. Mol Microbiol.

[37] Householder TC, Belli WA, Lissenden S, Cole JA, Clark VL (1999). cis- and trans-acting elements involved in regulation of aniA,
the gene encoding the major anaerobically induced outer membrane protein in
Neisseria gonorrhoeae.. J Bacteriol.

[38] Newcombe J, Eales-Reynolds LJ, Wootton L, Gorringe AR, Funnell SG (2004). Infection with an avirulent phoP mutant of Neisseria meningitidis
confers broad cross-reactive immunity.. Infect Immun.

[39] Tzeng YL, Zhou X, Bao S, Zhao S, Noble C (2006). Autoregulation of the MisR/MisS two-component signal transduction
system in Neisseria meningitidis.. J Bacteriol.

[40] Tzeng YL, Datta A, Ambrose K, Lo M, Davies JK (2004). The MisR/MisS two-component regulatory system influences inner
core structure and immunotype of lipooligosaccharide in Neisseria
meningitidis.. J Biol Chem.

[41] Jamet A, Rousseau C, Monfort JB, Frapy E, Nassif X (2009). A two-component system is required for colonization of host cells
by meningococcus.. Microbiology.

[42] Oshima T, Aiba H, Masuda Y, Kanaya S, Sugiura M (2002). Transcriptome analysis of all two-component regulatory system
mutants of Escherichia coli K-12.. Mol Microbiol.

[43] Janiak-Spens F, Sparling DP, West AH (2000). Novel role for an HPt domain in stabilizing the phosphorylated
state of a response regulator domain.. J Bacteriol.

[44] Larson JA, Higashi DL, Stojiljkovic I, So M (2002). Replication of Neisseria meningitidis within epithelial cells
requires TonB-dependent acquisition of host cell iron.. Infect Immun.

[45] Perkins-Balding D, Ratliff-Griffin M, Stojiljkovic I (2004). Iron transport systems in Neisseria meningitidis.. Microbiol Mol Biol Rev.

[46] Stojiljkovic I, Hwa V, de Saint Martin L, O'Gaora P, Nassif X (1995). The Neisseria meningitidis haemoglobin receptor: its role in iron
utilization and virulence.. Mol Microbiol.

[47] Leighton MP, Kelly DJ, Williamson MP, Shaw JG (2001). An NMR and enzyme study of the carbon metabolism of Neisseria
meningitidis.. Microbiology.

[48] Exley RM, Goodwin L, Mowe E, Shaw J, Smith H (2005). Neisseria meningitidis lactate permease is required for
nasopharyngeal colonization.. Infect Immun.

[49] Lee EH, Shafer WM (1999). The farAB-encoded efflux pump mediates resistance of gonococci to
long-chained antibacterial fatty acids.. Mol Microbiol.

[50] Hotopp JC, Grifantini R, Kumar N, Tzeng YL, Fouts D (2006). Comparative genomics of Neisseria meningitidis: core genome,
islands of horizontal transfer and pathogen-specific genes.. Microbiology.

[51] Colicchio R, Ricci S, Lamberti F, Pagliarulo C, Pagliuca C (2009). The meningococcal ABC-Type L-glutamate transporter GltT is
necessary for the development of experimental meningitis in
mice.. Infect Immun.

[52] Pagliarulo C, Salvatore P, De Vitis LR, Colicchio R, Monaco C (2004). Regulation and differential expression of gdhA encoding
NADP-specific glutamate dehydrogenase in Neisseria meningitidis clinical
isolates.. Mol Microbiol.

[53] Lo H, Tang CM, Exley RM (2009). Mechanisms of avoidance of host immunity by Neisseria
meningitidis and its effect on vaccine development.. Lancet Infect Dis.

[54] Turner DP, Marietou AG, Johnston L, Ho KK, Rogers AJ (2006). Characterization of MspA, an immunogenic autotransporter protein
that mediates adhesion to epithelial and endothelial cells in Neisseria
meningitidis.. Infect Immun.

[55] van Ulsen P, Adler B, Fassler P, Gilbert M, van Schilfgaarde M (2006). A novel phase-variable autotransporter serine protease, AusI, of
Neisseria meningitidis.. Microbes Infect.

[56] Paruchuri DK, Seifert HS, Ajioka RS, Karlsson KA, So M (1990). Identification and characterization of a Neisseria gonorrhoeae
gene encoding a glycolipid-binding adhesin.. Proc Natl Acad Sci U S A.

[57] Capecchi B, Adu-Bobie J, Di Marcello F, Ciucchi L, Masignani V (2005). Neisseria meningitidis NadA is a new invasin which promotes
bacterial adhesion to and penetration into human epithelial
cells.. Mol Microbiol.

[58] Scarselli M, Serruto D, Montanari P, Capecchi B, Adu-Bobie J (2006). Neisseria meningitidis NhhA is a multifunctional trimeric
autotransporter adhesin.. Mol Microbiol.

[59] Serruto D, Adu-Bobie J, Scarselli M, Veggi D, Pizza M (2003). Neisseria meningitidis App, a new adhesin with autocatalytic
serine protease activity.. Mol Microbiol.

[60] McNeil G, Virji M, Moxon ER (1994). Interactions of Neisseria meningitidis with human
monocytes.. Microb Pathog.

[61] Anjum MF, Stevanin TM, Read RC, Moir JW (2002). Nitric oxide metabolism in Neisseria
meningitidis.. J Bacteriol.

[62] Seib KL, Tseng HJ, McEwan AG, Apicella MA, Jennings MP (2004). Defenses against oxidative stress in Neisseria gonorrhoeae and
Neisseria meningitidis: distinctive systems for different
lifestyles.. J Infect Dis.

[63] Dunn KL, Farrant JL, Langford PR, Kroll JS (2003). Bacterial [Cu,Zn]-cofactored superoxide dismutase
protects opsonized, encapsulated Neisseria meningitidis from phagocytosis by
human monocytes/macrophages.. Infect Immun.

[64] Cardinale JA, Clark VL (2000). Expression of AniA, the major anaerobically induced outer
membrane protein of Neisseria gonorrhoeae, provides protection against
killing by normal human sera.. Infect Immun.

[65] Leuzzi R, Serino L, Scarselli M, Savino S, Fontana MR (2005). Ng-MIP, a surface-exposed lipoprotein of Neisseria gonorrhoeae,
has a peptidyl-prolyl cis/trans isomerase (PPIase) activity and is involved
in persistence in macrophages.. Mol Microbiol.

[66] Schneider MC, Exley RM, Ram S, Sim RB, Tang CM (2007). Interactions between Neisseria meningitidis and the complement
system.. Trends Microbiol.

[67] Jarva H, Ram S, Vogel U, Blom AM, Meri S (2005). Binding of the complement inhibitor C4bp to serogroup B Neisseria
meningitidis.. J Immunol.

[68] Madico G, Ngampasutadol J, Gulati S, Vogel U, Rice PA (2007). Factor H binding and function in sialylated pathogenic neisseriae
is influenced by gonococcal, but not meningococcal, porin.. J Immunol.

[69] Oriente F, Scarlato V, Delany I (2010). Expression of factor H binding protein of meningococcus responds
to oxygen limitation through a dedicated FNR-regulated
promoter.. J Bacteriol.

[70] Lewis LA, Ngampasutadol J, Wallace R, Reid JE, Vogel U (2010). The meningococcal vaccine candidate neisserial surface protein A
(NspA) binds to factor H and enhances meningococcal resistance to
complement.. PLoS Pathog.

[71] van Ulsen P, van Alphen L, ten Hove J, Fransen F, van der Ley P (2003). A Neisserial autotransporter NalP modulating the processing of
other autotransporters.. Mol Microbiol.

[72] Ait-Tahar K, Wooldridge KG, Turner DP, Atta M, Todd I (2000). Auto-transporter A protein of Neisseria meningitidis: a potent
CD4+ T-cell and B-cell stimulating antigen detected by expression
cloning.. Mol Microbiol.

[73] Peiser L, Makepeace K, Pluddemann A, Savino S, Wright JC (2006). Identification of Neisseria meningitidis nonlipopolysaccharide
ligands for class A macrophage scavenger receptor by using a novel
assay.. Infect Immun.

[74] Pluddemann A, Hoe JC, Makepeace K, Moxon ER, Gordon S (2009). The macrophage scavenger receptor A is host-protective in
experimental meningococcal septicaemia.. PLoS Pathog.

[75] Stohl EA, Criss AK, Seifert HS (2005). The transcriptome response of Neisseria gonorrhoeae to hydrogen
peroxide reveals genes with previously uncharacterized roles in oxidative
damage protection.. Mol Microbiol.

[76] Tidhar A, Flashner Y, Cohen S, Levi Y, Zauberman A (2009). The NlpD lipoprotein is a novel Yersinia pestis virulence factor
essential for the development of plague.. PLoS ONE.

[77] Feavers IM, Pizza M (2009). Meningococcal protein antigens and vaccines.. Vaccine.

[78] Giuliani MM, Adu-Bobie J, Comanducci M, Arico B, Savino S (2006). A universal vaccine for serogroup B
meningococcus.. Proc Natl Acad Sci U S A.

[79] Pizza M, Scarlato V, Masignani V, Giuliani MM, Arico B (2000). Identification of vaccine candidates against serogroup B
meningococcus by whole-genome sequencing.. Science.

[80] Metruccio MM, Pigozzi E, Roncarati D, Berlanda Scorza F, Norais N (2009). A novel phase variation mechanism in the meningococcus driven by
a ligand-responsive repressor and differential spacing of distal promoter
elements.. PLoS Pathog.

[81] Seib KL, Oriente F, Adu-Bobie J, Montanari P, Ferlicca F (2010). Influence of serogroup B meningococcal vaccine antigens on growth
and survival of the meningococcus in vitro and in ex vivo and in vivo models
of infection.. Vaccine.

[82] Muzzi A, Masignani V, Rappuoli R (2007). The pan-genome: towards a knowledge-based discovery of novel
targets for vaccines and antibacterials.. Drug Discov Today.

[83] Yu NY, Wagner JR, Laird MR, Melli G, Rey S (2010). PSORTb 3.0: improved protein subcellular localization prediction
with refined localization subcategories and predictive capabilities for all
prokaryotes.. Bioinformatics.

[84] Rey S, Acab M, Gardy JL, Laird MR, deFays K (2005). PSORTdb: a protein subcellular localization database for
bacteria.. Nucleic Acids Res.

[85] Exley RM, Shaw J, Mowe E, Sun YH, West NP (2005). Available carbon source influences the resistance of Neisseria
meningitidis against complement.. J Exp Med.

[86] Carpenter BM, Whitmire JM, Merrell DS (2009). This is not your mother's repressor: the complex role of fur
in pathogenesis.. Infect Immun.

[87] Joseph B, Schneiker-Bekel S, Schramm-Gluck A, Blom J, Claus H (2010). Comparative genome biology of a serogroup B carriage and disease
strain supports a polygenic nature of meningococcal
virulence.. J Bacteriol.

[88] Hughes TR, Mao M, Jones AR, Burchard J, Marton MJ (2001). Expression profiling using microarrays fabricated by an ink-jet
oligonucleotide synthesizer.. Nat Biotechnol.

[89] Charbonnier Y, Gettler B, Francois P, Bento M, Renzoni A (2005). A generic approach for the design of whole-genome oligoarrays,
validated for genomotyping, deletion mapping and gene expression analysis on
Staphylococcus aureus.. BMC Genomics.

[90] Saal LH, Troein C, Vallon-Christersson J, Gruvberger S, Borg A (2002). BioArray Software Environment (BASE): a platform for
comprehensive management and analysis of microarray data.. Genome Biol.

[91] Storey JD, Tibshirani R (2003). Statistical significance for genomewide studies.. Proc Natl Acad Sci U S A.

[92] Aryee MJ, Gutierrez-Pabello JA, Kramnik I, Maiti T, Quackenbush J (2009). An improved empirical bayes approach to estimating differential
gene expression in microarray time-course data: BETR (Bayesian Estimation of
Temporal Regulation).. BMC Bioinformatics.

[93] Giancarlo R, Scaturro D, Utro F (2008). Computational cluster validation for microarray data analysis:
experimental assessment of Clest, Consensus Clustering, Figure of Merit, Gap
Statistics and Model Explorer.. BMC Bioinformatics.

[94] Saeed AI, Sharov V, White J, Li J, Liang W (2003). TM4: a free, open-source system for microarray data management
and analysis.. Biotechniques.

[95] Ieva R, Alaimo C, Delany I, Spohn G, Rappuoli R (2005). CrgA is an inducible LysR-type regulator of Neisseria
meningitidis, acting both as a repressor and as an activator of gene
transcription.. J Bacteriol.

