# Supplementary figures and images for: Transcriptome Analysis of Neisseria meningitidis in Human Whole Blood and Mutagenesis Studies Identify Virulence Factors Involved in Blood Survival
Source: PLoS Pathog. 2011 May 5;7(5):e1002027. doi: 10.1371/journal.ppat.1002027 (PMC3088726; doi:10.1371/journal.ppat.1002027)

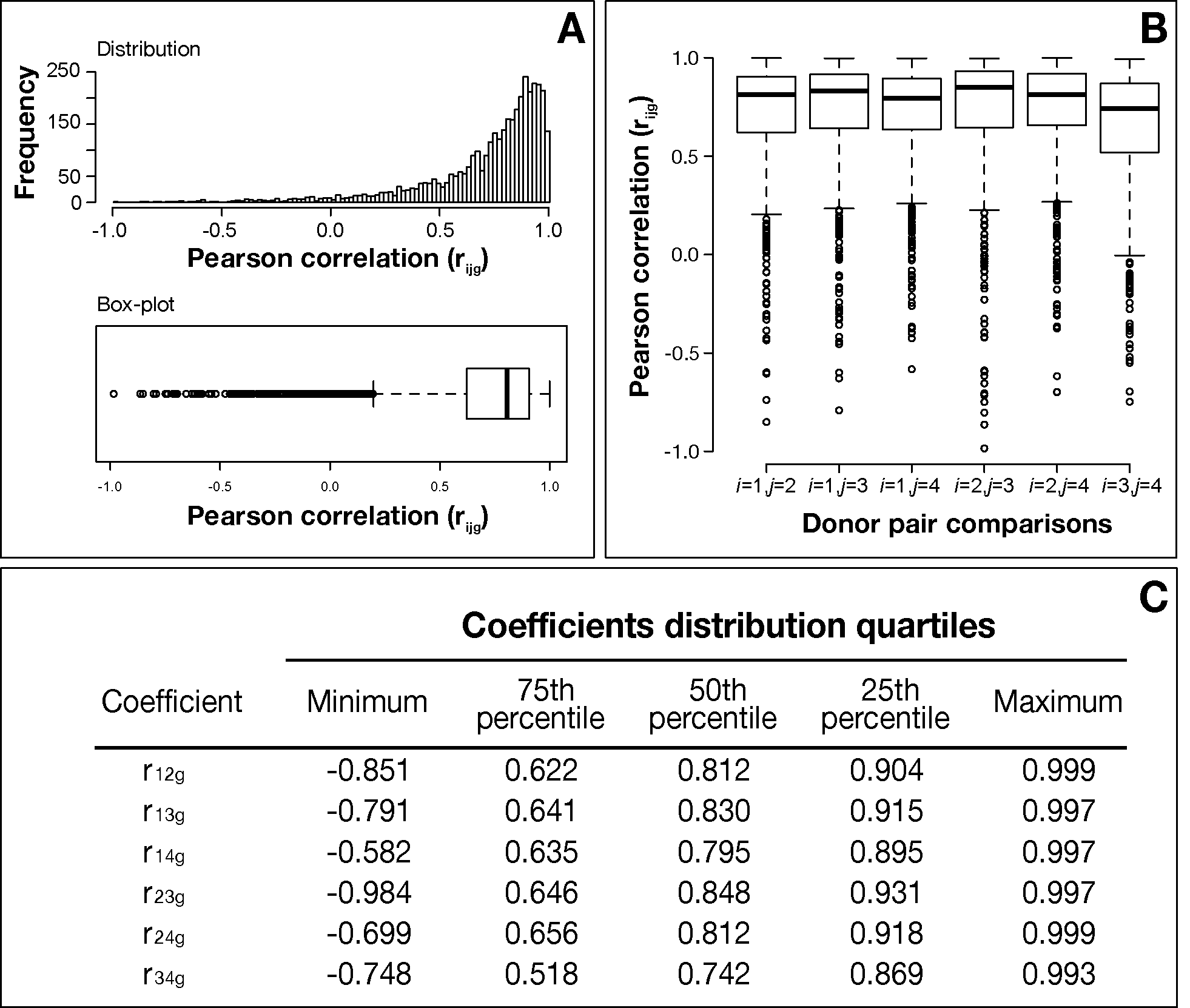

Supplement: Figure S1 — Statistical analysis of the correlation between donor samples. (A) Top panel, distribution of Pearson correlation rijg of gene expression profiles (g) between donor samples i and j bottom panel, the same distribution results shown as a box-plot. Typically genes show a good reproducibility (3/4 of the comparisons have r > 0.6). (B) Same analysis as performed in A but comparing pairs of data sets between the single blood donors shown in single box-plots. (C) Quartile statistics values are reported in the table for pairs of donors shown in panel B. (TIF) [file ppat.1002027.s001.tif]

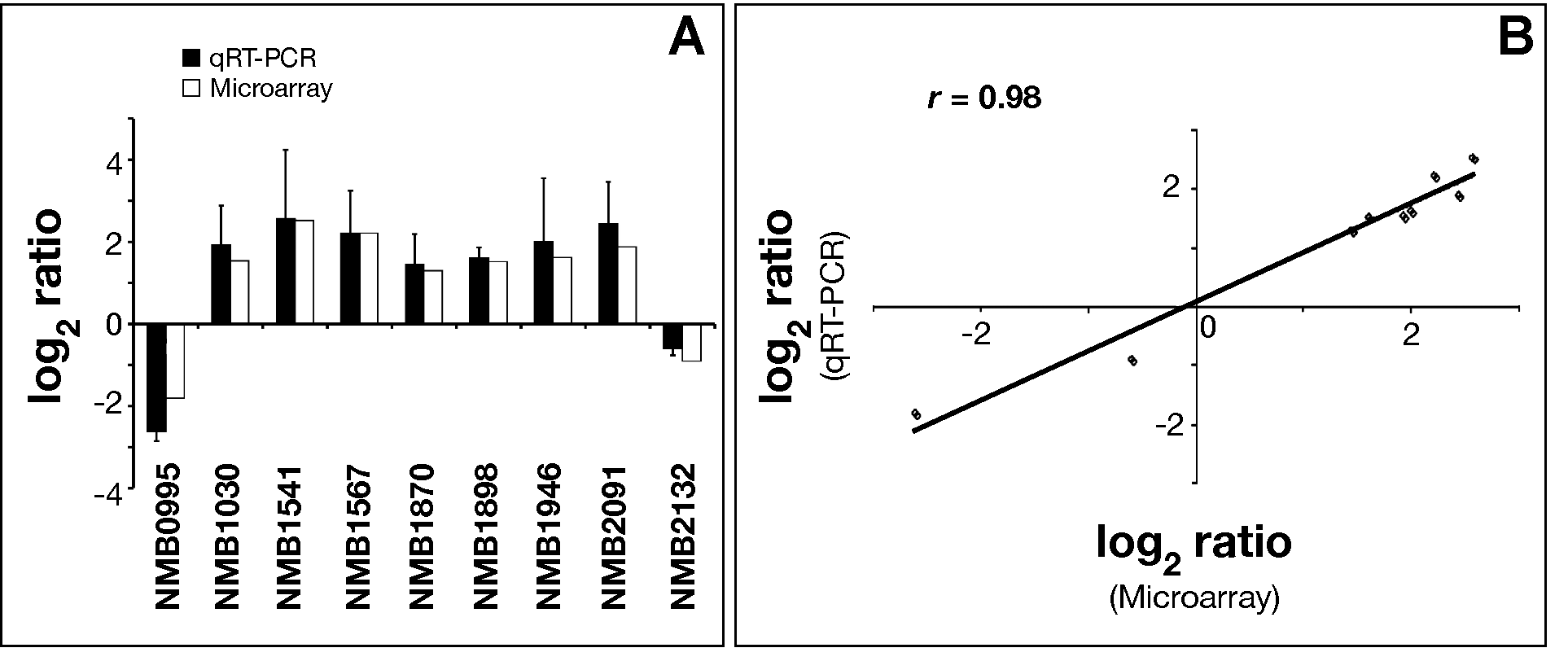

Supplement: Figure S2 — Validation of microarray data by qRT-PCR. (A) Comparison of microarray (white bars) and qRT-PCR (black bars) fold change results for 9 selected genes. Fold change qRT-PCR ratios represent the difference in transcript abundance/signal for these genes after 45 minutes of incubation in human whole blood as compared to time 0. qRT-PCR normalization data was done using 16S rRNA as a reference gene. The qRT-PCR and microarray data are represented as the mean of gene expression of four independent experiments. (B) Correlation analysis of microarray and qRT-PCR transcript measurements for the nine selected genes shown in panel A. The qRT-PCR and microarray log2 values were plotted and the coefficient of correlation was calculated, r = 0.98. (TIF) [file ppat.1002027.s002.tif]

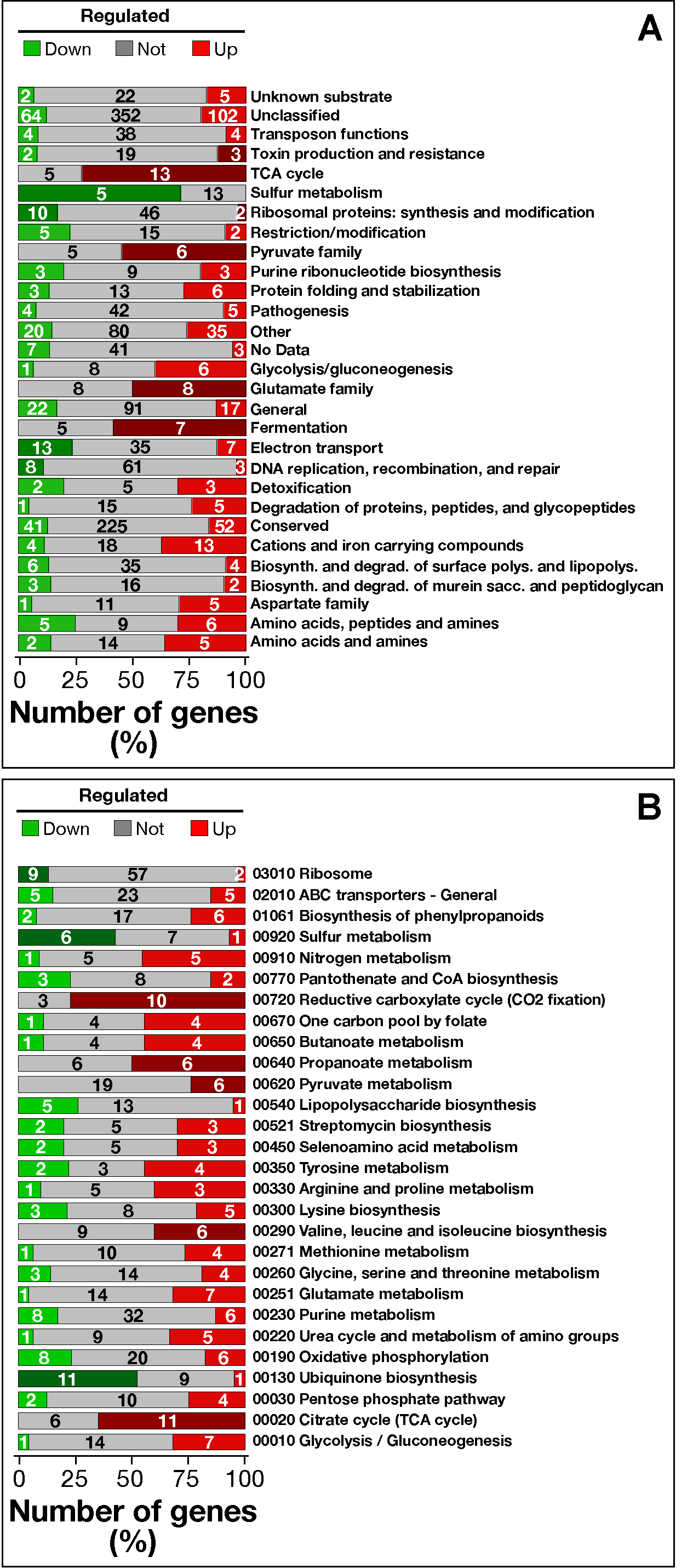

Supplement: Figure S3 — Distribution of differentially regulated genes within TIGRFAM sub-roles and KEGG pathways. (A) TIGR families sub-roles and (B) KEGG pathways. The groups with five or more differentially regulated genes are represented. Darker bars indicate the most relevant enrichments among the included groups (Fisher s exact test p-value < 0.05). (TIF) [file ppat.1002027.s003.tif]

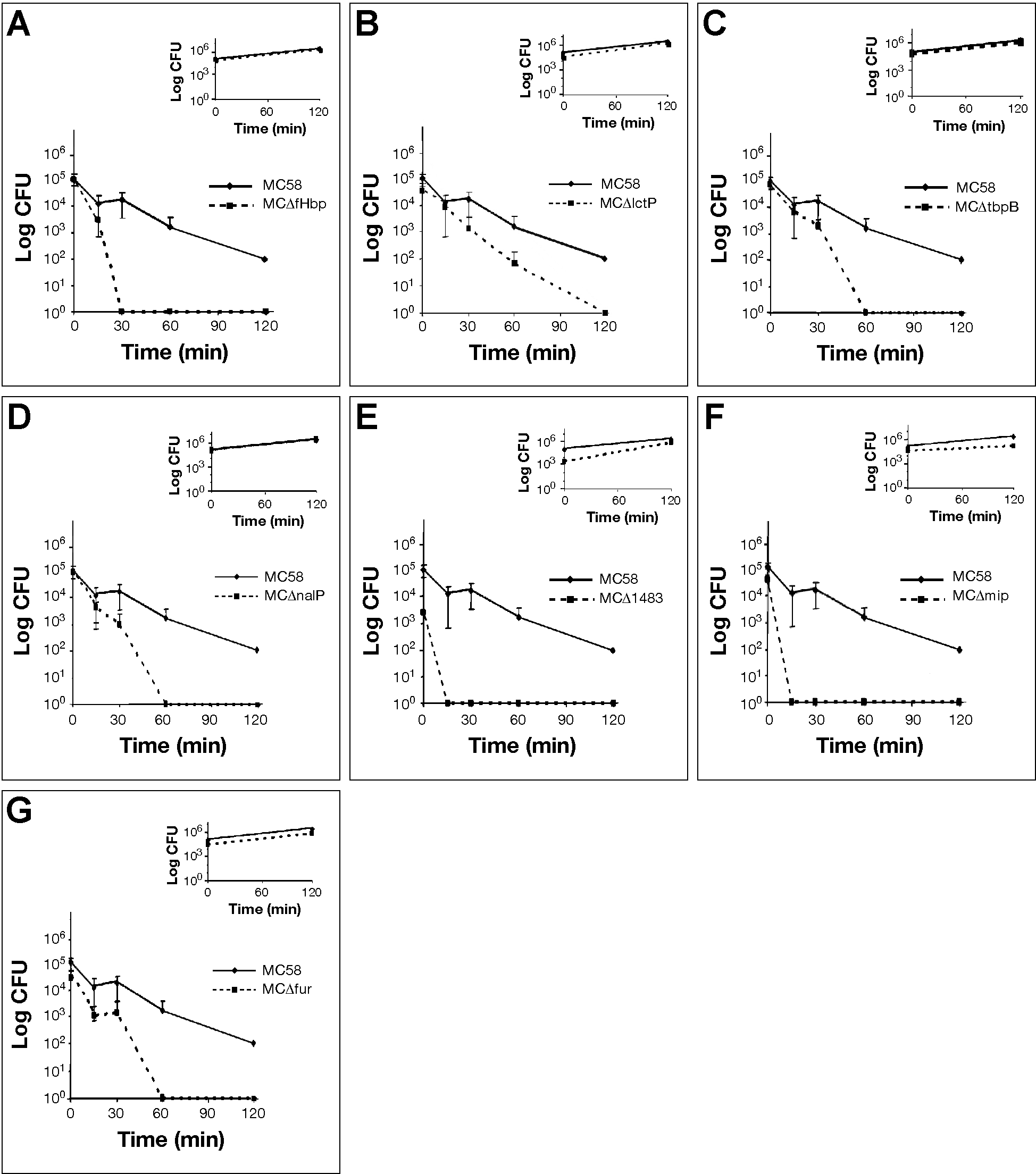

Supplement: Figure S5 — Survival of MC58 wild-type and deletion mutant strains in the ex vivo whole blood model using a second blood donor. Deletion mutants of the selected up-regulated genes were tested for survival using the ex vivo whole human blood model over a time course of 120 minutes. In each panel the phenotype of the specific mutant is compared to MC58 wild-type strain. The MCDfHbp deletion mutant was used as a control. The insets of each panel represent the growth control in GC medium for the same time course of incubation as done with whole blood. (TIF) [file ppat.1002027.s005.tif]
