# Supplementary material for: Transcriptome Analysis of Neisseria meningitidis in Human Whole Blood and Mutagenesis Studies Identify Virulence Factors Involved in Blood Survival
Source: PLoS Pathog. 2011 May 5;7(5):e1002027. doi: 10.1371/journal.ppat.1002027 (PMC3088726; doi:10.1371/journal.ppat.1002027)
Supplement: Table S3 — Primers used in this study. (DOC) [file ppat.1002027.s008.doc]

**Table S3. Primers used in this study**

| **Primer** | **Sequence 5’- 3’** | **Restriction sites^b^** |
| --- | --- | --- |
| **qRT-PCR** | | |
| 16S_Fw | -ACGGAGGGTGCGAGCGTTAATC- |  |
| 16S_Rv | -CTGCCTTCGCCTTCGGTATTCCT- |  |
| *NMB0995*_Fw | -TGTTTGCAGCACATAACCAAT- |  |
| *NMB0995*_Rv | -TGTACGGTTAAACGTGCCAT- |  |
| *NMB1030*_Fw | -AATCATCTTCGCCGCACT- |  |
| *NMB1030*_Rv | -GGCGTGATATTCGTCCACTT- |  |
| *NMB1541*_Fw | -TACGGAAATTCCCGACAAGCA- |  |
| *NMB1541*_Rv | -TTGTCAGCCTGTTCTTTCAGC- |  |
| *NMB1567*_Fw | -TCCCTGAAGCAAATGAAGGA- |  |
| *NMB1567*_Rv | -ACTTCCTGAGCCTGCTCTT- |  |
| *NMB1870*_Fw | -GACCATAAAGACAAAGGTTTG- |  |
| *NMB1870*_Rv | -GCTGACCTTGTCGTTCTTCA- |  |
| *NMB1898*_Fw | -TGACCAAAGACGACATCTAC- |  |
| *NMB1898*_Rv | -TATGGAAGTGGAACAGGTAAT- |  |
| *NMB1946*_Fw | -ACCGACTATGTACGCCCGAA- |  |
| *NMB1946*_Rv | -TTCGGTGATGTCCAGATTGT- |  |
| *NMB2091*_Fw | -ACGTTATGGCGTTGCGTAT- |  |
| *NMB2091*_Rv | -TGTAGCCTTTGGTTTGGTTG- |  |
| *NMB2132*_Fw | -ACAGAGGCAAAGGAAGATGC- |  |
| *NMB2132*_Rv | -CCATTGCCTGTATTTTCTTC- |  |
| **Deletion mutants** | | |
| *Up0035_Fw* | - gctctagaGCAGCTTCAGGATTCTGTGC- | XbaI |
| *Up0035_Rv* | - tcccccgggTTCAGCTCCTCTTTACGGGT- | SmaI |
| *Dn0035_Fw* | -tcccccgggGCCGCAAGCGTTCAGACGGT - | SmaI |
| *Dn0035_Rv* | - ccgctcgaGAAGGCTTTGCCCAAAATGC - | XhoI |
| *C0035_Fw* | - GGAACGCGCTTCAGGACATT - |  |
| *C0035_Rv* | - CCGAGATAACCTCCGATTGC - |  |
| *Upkat_Fw* | - gctctagaGCGTATGGTCGAGGATGTCT - | XbaI |
| *Upkat_Rv* | -tcccccgggGGGGGTAAACGGCTTACAGT - | SmaI |
| *Dnkat_Fw* | -tcccccgggGTAAGGGGGCATTATGTGGA - | SmaI |
| *Dnkat_Rv* | - ccgctcgaATAGGCTTTCCCGTTTGCTT - | XhoI |
| *Ckat_Fw* | - ACTGTAAGCCGTTTACCCCC - |  |
| *Ckat_Rv* | - TCCACATAATGCCCCCTTAC - |  |
| *UptbpB_Fw^1^* | - gctctagaGGAATGACGCGAACAGAAAC - | XbaI |
| *UptbpB_Rv* | -tcccccgggAGCCTGATTCACCAATGGATT- | SmaI |
| *DntbpB_Fw* | - tcccccgggGCCAACAGCCTGTGCGATA - | SmaI |
| *DntbpB_Rv^2^* | - ccgctcgaTTCAACCGGCACCAGCCTGTT- | XhoI |
| *CtbpB_Fw* | - AATCCATTGGTGAATCAGGCT - |  |
| *CtbpB_Rv* | - TATCGCACAGGCTGTTGGC - |  |
| *UplctP_Fw^1^* | - gctctagaGCCGCATCATCACTATCCTT - | XbaI |
| *UplctP_Rv* | - tcccccgggCGTAAATCAGCACTGCGGTA - | SmaI |
| *DnlctP_Fw* | - tcccccgggCGACAATTCGATGGTGAAAA - | SmaI |
| *DnlctP_Rv^2^* | - ccgctcgaAAATCTGCCGCTGTATTGCT - | XhoI |
| *ClctP_Fw* | - TACCGCAGTGCTGATTTACG - |  |
| *ClctP_Rv* | - TTTTCACCATCGAATTGTCG - |  |
| *Up0595_Fw* | - gctctagaCCATGCAAGACATTGCAAAA - | XbaI |
| *Up0595_Rv* | - tcccccgggATTTTGGGCATCATGGAATC - | SmaI |
| *Dn0595_Fw* | - tcccccgggAAAATTCGACCGCAGTATCG - | SmaI |
| *Dn0595_Rv* | -ccgctcgaGAGATGGGACAATTCGTCGTC - | XhoI |
| *C0595_Fw* | - GATTCCATGATGCCCAAAAT - |  |
| *C0595_Rv* | - CGATACTGCGGTCGAATTTT - |  |
| *UpnspA_Fw^1^* | - gctctagaTGTGAAGTGGGAAAGTGTTG - | XbaI |
| *UpnspA_Rv* | - tcccccgggTGCGCCTTATTCTGCAAACC - | SmaI |
| *DnnspA_Fw* | -tcccccgggGGTTCCTTTATGGTCAGTTAG - | SmaI |
| *DnnspA_Rv^2^* | - ccgctcgaGAACGCGTCCGGAAAATATG - | XhoI |
| *C_nspA_Fw* | - GGTTTGCAGAATAAGGCGCA - |  |
| *C_nspA_Rv* | - CTAACTGACCATAAAGGAACC - |  |
| *Upopc_Fw* | - gctctagaCGATGATGTTGTAGCGGA - | XbaI |
| *Upopc_Rv* | - tcccccgggGTCACTTTAAATGCCAAACC - | SmaI |
| *Dnopc_Fw* | - tcccccgggTGGATTGTAGTCGGATATG - | SmaI |
| *Dnopc_Rv* | - ccgctcgaCTATCGGAAATAACCGAAACC - | XhoI |
| *Copc_Fw* | - GGTTTGGCATTTAAAGTGAC - |  |
| *Copc_Rv* | - CATATCCGACTACAATCCA - |  |
| *Up1064_Fw* | - gctctagaATAAACCGAGCGGTTCTTGA - | XbaI |
| *Up1064_Rv* | - tcccccgggTCGCCGCCTAATTTTACTTC - | SmaI |
| *Dn1064_Fw* | - tcccccgggCGGTTTGCAATACTGGTTGA - | SmaI |
| *Dn1064_Rv* | - ccgctcgagCTGCTGCTGATTACCGGTTT - | XhoI |
| *C1064_Fw* | - GAAGTAAAATTAGGCGGCGA - |  |
| *C1064_Rv* | - TCAACCAGTATTGCAAACCG - |  |
| *Up1483_Fw^1^* | - gctctagaCGTTACAGCGGCAATTATTGC - | XbaI |
| *Up1483_Rv* | -tcccccgggCGCAGACAGTACAGATAGTAC- | SmaI |
| *Dn1483_Fw* | - tcccccgggATGTTCCGATATATAGCCTG - | SmaI |
| *Dn1483_Rv^2^* | - ccgctcgaCCCCTATTTTGTGGAACATC - | XhoI |
| *C1483_Fw* | - CCATCCGTTTCCATTGCAAAC - |  |
| *C1483_Rv* | - TGCGGACTGACCGTTTCATC - |  |
| *Up1786_Fw* | - gctctagaATGTCGCATTGTTCCAAACC - | XbaI |
| *Up1786_Rv* | -tcccccgggGGATGCTCATTACTTCCCCTTA- | SmaI |
| *Dn1786_Fw* | - tcccccgggTGGAAATTTTGTGAGGGATTC- | SmaI |
| *Dn1786_Rv* | - ccgctcgagCTTTACCCGTCAGGCTGGTT - | XhoI |
| *C1786_Fw* | - TAAGGGGAAGTAATGAGCATCC - |  |
| *C1786_Rv* | - GAATCCCTCACAAAATTTCCA - |  |
| *Up1840_Fw* | - gctctagaAACTGGCGGTCGTTCATATC - | XbaI |
| *Up1840_Rv* | - tcccccgggCTGCCCATGTTTTCTCCTTG - | SmaI |
| *Dn1840_Fw* | - tcccccgggGCTTGAGCCTCTTTCAGACG - | SmaI |
| *Dn1840_Rv* | - ccgctcgagAGGCTCGACGAAATCAAAAA - | XhoI |
| *C1840_Fw* | - CAAGGAGAAAACATGGGCAG - |  |
| *C1840_Rv* | - CGTCTGAAAGAGGCTCAAGC - |  |
| *Up1946_Fw* | - gctctagaGATGATGGTACGGCGTTTG - | XbaI |
| *Up1946_Rv* | - tcccccgggGGCAGTCGTATAAAATGATG - | SmaI |
| *Dn1946_Fw* | - tcccccgggCATTTTCTCCTGATGTTGTG - | SmaI |
| *Dn1946_Rv* | - ccgctcgagCTTCGTCATCCTGATGATTG - | XhoI |
| *C1946_Fw* | -CATCATTTTATACGACTGCC - |  |
| *C1946_Rv* | - CCACAACATCAGGAGAAAAT - |  |
| **Complementing strains** | | |
| NspAcmpF | -gggaattccatATGAAAAAAGCACTTGCCACAC- | NdeI |
| NspAcmpR | -ccaatgcatTCAGAATTTGACGCGCACAC- | NsiI |
| 1483cmpF | -gggaattccatATGTTGAAACAAACGACAC- | NdeI |
| 1483cmpR | -ccaatgcatTCAGAACGCGATATAGCTGTT- | NsiI |
| 1567cmpF | -gggaattccatATGAACACCATTTTCAAAATC- | NdeI |
| 1567cmpR | -ccaatgcatTTAATTTACTTTTTTGATGTCGAC- | NsiI |
| **Sequencing of deletion mutants** | | |
| Up*fHbp*_Fw^1^ | -CCAGCCAGGCGCATAC- |  |
| Dn*fHbp*_Rv^2^ | -CAGCGTATCGAACCATGC- |  |
| Upmip_Fw^1^ | -TCGGACGGCTTCAGGA- |  |
| Dnmip_Rv^2^ | -GACACGGTTTCCCTCGA- |  |
| Up*fur*_Fw^1^ | -GAGCGGTGTCATGTGTGTTCC- |  |
| Dn*fur*_Rv^2^ | -GAATGCGCGTACCCCATTTCG- |  |
| Up*nalP*_Fw^1^ | -CATATGCGAACGACCCCAACCTTCC- |  |
| Dn*nalP*_Rv^2^ | -TGCATGCATTCAGAACCGGTAGCCTACG- |  |
| SeqKan Rv^3^ | -CGCTTCATAGAGTAATTCTG- |  |
| SeqKan Fw^4^ | -GACTTACTGGGGATCAAGC- |  |
| SeqEry Rv^3^ | -CATGACGAATCCCTCCTTC- |  |
| SeqEry Fw^4^ | -CGTTACTAAAGGGAATGGAG- |  |
| **Southern blot analysis** | | |
| SB_Ery Fw | -GAAGGAGGGATTCGTCATG- |  |
| SB_Ery Rv | -CTCCATTCCCTTTAGTAACG- |  |
| SB_Kan Fw | -CTATAGAATGGGCAAAGCAT- |  |
| SB_Kan Rv | -GCTTGATCCCCAGTAAGTC- |  |

^a^ Capital letters correspond to nucleotides of the meningococcal sequence and small letters correspond to nucleotides added for cloning reasons.

^b^ Enzymes for which the restriction sites are present in the sequence of the primer, added for cloning reasons.

^1^ and ^2^ indicates the pair of primers used to generate the PCR fragments for sequencing.

^3^ and ^4^ indicates the primers used to sequence the flanking regions of the recombination site of the deletion mutants and to determine the antibiotic resistance cassette orientation (see Figure S4).
