# Supplementary material for: Transcriptome Analysis of Neisseria meningitidis in Human Whole Blood and Mutagenesis Studies Identify Virulence Factors Involved in Blood Survival
Source: PLoS Pathog. 2011 May 5;7(5):e1002027. doi: 10.1371/journal.ppat.1002027 (PMC3088726; doi:10.1371/journal.ppat.1002027)
Supplement: Table S4 — Plasmids used in this study. (DOC) [file ppat.1002027.s009.doc]

**Table S4.** **Plasmids used in this study.**

| Plasmid | Relevant characteristics | Reference or source |
| --- | --- | --- |
| Deletion mutants | | |
| pBluescript (pBS) | Cloning vector, Amp^r^ | Stratagene |
| pBS-UD*0035*_Ery | Construct for generating DELETION MUTANT of the *nmb0035* gene, Amp^r^Ery^r^ | This study |
| pBS-UD*kat*_Ery | Construct for generating DELETION MUTANT of the *kat (NMB0216)* gene, Amp^r^Ery^r^ | This study |
| pBS-UD*tbpB*_Ery | Construct for generating DELETION MUTANT of the *tbpB* (*NMB0460*) gene, Amp^r^Ery^r^ | This study |
| pBS-UD*lctP*_Ery | Construct for generating DELETION MUTANT of the *lctP* (*NMB0543*) gene, Amp^r^Ery^r^ | This study |
| pBS-UD*0595*_Ery | Construct for generating DELETION MUTANT of the *lctP* (*NMB0595*) gene, Amp^r^Ery^r^ | This study |
| pBS-UD*nspA*_Ery | Construct for generating DELETION MUTANT of the *nspA* (*NMB0663*) gene, Amp^r^Ery^r^ | This study |
| pBS-UD*opc*_Ery | Construct for generating DELETION MUTANT of the *opc (NMB1053)* gene, Amp^r^Ery^r^ | This study |
| pBS-UD*1064*_Ery | Construct for generating DELETION MUTANT of the *NMB1064*  gene, Amp^r^Ery^r^ | This study |
| pBS-UD*1483*_Ery | Construct for generating DELETION MUTANT of the *NMB1483* gene, Amp^r^Ery^r^ | This study |
| pBS-UD*mip_*Ery | Construct for generating DELETION MUTANT of the *mip* (*NMB1567*) gene, Amp^r^Ery^r^ | Leuzzi *et al.,* 2005 |
| pBS-UD*1786*_Ery | Construct for generating DELETION MUTANT of the *NMB1786* gene, Amp^r^Ery^r^ | This study |
| pBS-UD*1840_*Ery | Construct for generating DELETION MUTANT of the *NMB1840* gene, Amp^r^Ery^r^ | This study |
| pBS-UD*fHbp*_Ery | Construct for generating DELETION MUTANT of the *fHbp (NMB1870)* gene, Amp^r^Ery^r^ | Seib *et al.,* 2009 |
| pBS-UD*1946_*Ery | Construct for generating DELETION MUTANT of the *NMB1946* gene, Amp^r^Ery^r^ | This study |
| pBS-UD*nalP*_Kan | Construct for generating DELETION MUTANT of the *nalP* *(NMB1969)* gene, Amp^r^Kan^r^ | This study |
| Complementing strains | | |
| pCom-pRBS | Derivative of pSLcomCmr with tac promoter and downstream ribosome binding site. Cm^r^Amp^r^ | Ieva *et al.,* 2005 |
| pComppRBS-*nspA* | Construct to generate complementing strain for *nspA* gene (*NMB0663*) in 95N477 strain, Cm^r^Amp^r^ | This study |
| pCompRBS-*1483* | Construct to generate complementing strain for the gene *NMB1483* in MC58, Cm^r^Amp^r^ | This study |
| pCompRBS-*mip* | Construct to generate complementing strain for *mip* gene (*NMB1567*) in MC58, Cm^r^Amp^r^ | This study |

Amp^r^ = ampicillin resistance cassette

Cm^r^= chloramphenicol resistance cassette

Ery^r^ = erythromycin resistance cassette

Kan*^r^*=kanamycin resistance cassette
