# Supplementary material for: Transcriptome Analysis of Neisseria meningitidis in Human Whole Blood and Mutagenesis Studies Identify Virulence Factors Involved in Blood Survival
Source: PLoS Pathog. 2011 May 5;7(5):e1002027. doi: 10.1371/journal.ppat.1002027 (PMC3088726; doi:10.1371/journal.ppat.1002027)
Supplement: Table S5 — Nm wild-type, deletion mutants and complementing strains used in this study. (DOC) [file ppat.1002027.s010.doc]

**Table S5. Nm wild-type, deletion mutants and complementing strains used in this study.**

| **Name** | **Relevant characteristics** | **Reference or source** |
| --- | --- | --- |
| *Neisseria meningitidis* | | |
| MC58 | Clinical isolate, B:15:P1.7,16b, cpx 32, ST 74 | Tettelin *et al*., 2000 |
| MCΔ*fur* | *fur* (*NMB0205*) deletion mutant of MC58, Ery^r^ | Delany *et al*., 2006 |
| MCΔ*kat* | *kat (NMB0216)* deletion mutant of MC58, Ery^r^ | This study |
| MCΔt*bpB* | *tbpB* (*NMB0460*) deletion mutant of MC58, Ery^r^ | This study |
| MCΔ*lctP* | *lctP* (*NMB0543*) deletion mutant of MC58, Ery^r^ | This study |
| MCΔ*nspA* | *nspA* (*NMB0663*) deletion mutant of MC58, Ery^r^ | This study |
| MCΔ*opc* | *opc (NMB1053*) deletion mutant of MC58, Ery^r^ | This study |
| MCΔ1*483* | *NMB1483* deletion mutant of MC58, Ery^r^ | This study |
| MCΔ*mip* | *mip* (*NMB1567*) deletion mutant of MC58, Ery^r^ | This study |
| MCΔ*fHhp* | *fHbp* (*NMB1870*) deletion mutant of MC58, Ery^r^ | Seib *et al*., 2009 |
| MCΔ*nalP* | *nalP* (*NMB1969*) deletion mutant of MC58, Kan^r^ | Serruto *et al.,* 2010 |
| MCΔ*0035* | *NMB0035* deletion mutant of MC58, Ery^r^ | This study |
| MCΔ*0595* | *NMB0595* deletion mutant of MC58, Ery^r^ | This study |
| MCΔ*1064* | *NMB1064* deletion mutant of MC58, Ery^r^ | This study |
| MCΔ*1786* | *NMB1786* deletion mutant of MC58, Ery^r^ | This study |
| MCΔ*1840* | *NMB1840* deletion mutant of MC58, Ery^r^ | This study |
| MCΔ*1946* | *NMB1946* deletion mutant of MC58, Ery^r^ | This study |
| MCΔ−*Cfur* | MCΔ*fur* complemented with *fur,* Kan^r^Cm^r^ | Delany *et al.*, 2006 |
| MCΔ−*C1483* | MCΔ1*483* complemented with *1483,* Ery^r^Cm^r^ | This study |
| MCΔ−*Cmip* | MCΔ*mip* complemented with *mip,* Ery^r^Cm^r^ | This study |

| 95N477 | clinical isolate, B:2a:P1.2, cpx 11, ST 475 | Jelfs *et al.,* 2000 |
| --- | --- | --- |
| 95Δ*kat* | *kat (NMB0216)* deletion mutant of 95N477, Ery^r^ | This study |
| 95Δ*tbpB* | *tbpB (NMB0460)* deletion mutant of 95N477, Ery^r^ | This study |
| 95Δ*lctP* | *lctP (NMB0543)* deletion mutant of 95N477, Ery^r^ | This study |
| 95Δ*nspA* | *nspA (NMB0663)* deletion mutant of 95N477, Ery^r^ | This study |
| 95Δ*1483* | *NMB1483* deletion mutant of 95N477, Ery^r^ | This study |
| 95Δ*fHbp* | *fHbp (NMB1870)* deletion mutant of 95N477, Ery^r^ | This study |
| 95Δ*0035* | *NMB0035* deletion mutant of 95N477, Ery^r^ | This study |
| 95Δ*1786* | *NMB1786* deletion mutant of 95N477, Ery^r^ | This study |
| 95Δ*1840* | *NMB1840* deletion mutant of 95N477, Ery^r^ | This study |
| 95Δ*1946* | *NMB1946* deletion mutant of 95N477, Ery^r^ | This study |
| 95Δ−*CnspA* | *95*Δ*nspA* complemented with *nspA*, Ery^r^Cm^r^ | This study |

Cm^r^= chloramphenicol resistance cassette

Ery^r^ = erythromycin resistance cassette

Kan^r^=kanamycin resistance cassette
